# Lethal (2) giant discs (Lgd)/CC2D1 is required for the full activity of the ESCRT machinery

**DOI:** 10.1186/s12915-020-00933-x

**Published:** 2020-12-22

**Authors:** Miriam Baeumers, Kristina Ruhnau, Thomas Breuer, Hendrik Pannen, Bastian Goerlich, Anna Kniebel, Sebastian Haensch, Stefanie Weidtkamp-Peters, Lutz Schmitt, Thomas Klein

**Affiliations:** 1 Institute of Genetics, Heinrich-Heine-Universitaet Duesseldorf, Universitaetsstr. 1, 40225 Duesseldorf, Germany; 2 Center of Advanced Imaging (CAi), Heinrich-Heine-Universitaet Duesseldorf, Universitaetsstr. 1, 40225 Duesseldorf, Germany; 3 Institute of Biochemistry I, Heinrich-Heine-Universitaet Duesseldorf, Universitaetsstr. 1, 40225 Duesseldorf, Germany

**Keywords:** Lethal (2) giant discs, Lgd, CC2D1A, CC2D1B, ESCRT, Shrub, CHMP4B, Notch pathway, Endosomal pathway

## Abstract

**Background:**

A major task of the endosomal sorting complex required for transport (ESCRT) machinery is the pinching off of cargo-loaded intraluminal vesicles (ILVs) into the lumen of maturing endosomes (MEs), which is essential for the complete degradation of transmembrane proteins in the lysosome. The ESCRT machinery is also required for the termination of signalling through activated signalling receptors, as it separates their intracellular domains from the cytosol. At the heart of the machinery lies the ESCRT-III complex, which is required for an increasing number of processes where membrane regions are abscised away from the cytosol. The core of ESCRT-III, comprising four members of the CHMP protein family, organises the assembly of a homopolymer of CHMP4, Shrub in *Drosophila*, that is essential for abscission. We and others identified the tumour-suppressor lethal (2) giant discs (Lgd)/CC2D1 as a physical interactor of Shrub/CHMP4 in *Drosophila* and mammals, respectively.

**Results:**

Here, we show that the loss of function of *lgd* constitutes a state of reduced activity of Shrub/CHMP4/ESCRT-III. This hypomorphic *shrub* mutant situation causes a slight decrease in the rate of ILV formation that appears to result in incomplete incorporation of Notch into ILVs. We found that the forced incorporation in ILVs of *lgd* mutant MEs suppresses the uncontrolled and ligand-independent activation of Notch. Moreover, the analysis of *Su(dx) lgd* double mutants clarifies their relationship and suggests that they are not operating in a linear pathway. We could show that, despite prolonged lifetime, the MEs of *lgd* mutants have a similar ILV density as wild-type but less than *rab7* mutant MEs, suggesting the rate in *lgd* mutants is slightly reduced. The analysis of the MEs of wild-type and mutant cells in the electron microscope revealed that the ESCRT-containing electron-dense microdomains of ILV formation at the limiting membrane are elongated, indicating a change in ESCRT activity. Since *lgd* mutants can be rescued to normal adult flies if extra copies of *shrub* (or its mammalian ortholog *CHMP4B*) are added into the genome, we conclude that the net activity of Shrub is reduced upon loss of *lgd* function. Finally, we show that, in solution, CHMP4B/Shrub exists in two conformations. LGD1/Lgd binding does not affect the conformational state of Shrub, suggesting that Lgd is not a chaperone for Shrub/CHMP4B.

**Conclusion:**

Our results suggest that Lgd is required for the full activity of Shrub/ESCRT-III. In its absence, the activity of the ESCRT machinery is reduced. This reduction causes the escape of a fraction of cargo, among it Notch, from incorporation into ILVs, which in turn leads to an activation of this fraction of Notch after fusion of the ME with the lysosome. Our results highlight the importance of the incorporation of Notch into ILV not only to assure complete degradation, but also to avoid uncontrolled activation of the pathway.

## Background

The evolutionary conserved ESCRT machinery regulates the incorporation of transmembrane proteins (TMPs) into intraluminal vesicles (ILVs) of the maturing endosomes (MEs) (reviewed in [1]). ILV formation is essential for the complete degradation of TMPs in the lysosome, since it imports their intracellular domains (ICDs), which after endocytosis initially protrude into the cytosol, into the lumen of the ME. It is also required to terminate signalling by activated receptors, as it separates their ICDs from the cytosol. As a consequence of ESCRT activity, MEs accumulate ILVs during maturation and are recognised in the transmission electron microscope (TEM) as multi-vesicular bodies (MVBs).

The ESCRT machinery comprises five in sequence acting protein complexes, termed ESCRT-0, ESCRT-I, ESCRT-II, ESCRT-III and Vps4 [1]. ESCRT-0 till ESCRT-II concentrate ubiquitylated cargo at a spot of the limiting membrane (LM) of the ME, which is eventually pinched off as an ILV by the concerted action of ESCRT-III and Vps4. ESCRT-III assembles only on endosomal membranes from monomers that are recruited from the cytosol. It consists of four core subunits, termed Vps20, Shrub, Vps2 and Vps24 in *Drosophila*. All four have a high structural similarity and belong to the CHMP protein family. Vps20, activated by the previous acting ESCRT-II, initiates the polymerisation of Shrub monomers into a membrane-bound filament, which is eventually capped by Vps24 and Vps2. The short-living filament is disassembled by the AAA-ATPase Vps4 during abscission of the membrane. The mechanism of the abscission process is not well understood, but polymerisation of Shrub is essential. ESCRT-III and Vps4 mediate also other, topological similar membrane abscission events away from the cytosol in processes, such as plasma membrane repair, cytokinesis and the release of enveloped viruses from infected cells (e.g. Ebola and HIV, reviewed in [1]). The recruitment of ESCRT-III in these other processes requires different adaptors.

In *Drosophila*, the loss of function of ESCRT-I till ESCRT-III causes cell over-proliferation, uncontrolled activation of the Notch signalling pathway and extended signalling of other signalling pathways, such as the Dpp/BMP and Wg/Wnt pathways [2]. In addition, the apicobasal polarity of epithelia is lost [2, 3]. Therefore, the genes encoding members of ESCRT-I till ESCRT-III are classified as neoplastic tumour-suppressor genes.

Recent work showed that Shrub polymerises in a staggering array using complementary charged surfaces for electrostatic interactions [4]. The complementary electrostatic surfaces found in Shrub are conserved in all metazoans, suggesting a switch to electrostatic interactions in the evolution of metazoans [4]. The human genome contains three Shrub orthologs termed CHMP4A, B, C, encoded by separate genes.

Members of the conserved Lgd/CC2D1 protein family interact with Shrub/CHMP4B family members and are important for its activity [5–9]. In *Drosophila*, the loss of function of one of the founder of the family, termed Lethal (2) giant discs (Lgd), causes a ligand-independent constitutive activation of the Notch signalling pathway, which induces over-proliferation of the cells of the imaginal discs (reviewed in [10]). Moreover, its loss of function prolongs Dpp/BMP signalling in the female germline [11]. These phenotypes are caused by an endosomal defect that disturbs the trafficking of Notch and activated BMP receptors [10]. As a result, their degradation is delayed and the lifetime of the ME is increased [7]. *lgd* is present in the genomes of all metazoans and contains four repeats of the DM14 domain, followed by a C2 domain [12]. In mammals, two orthologs are present, LGD1, also named CC2D1B, and LGD2, also named CC2D1A [13, 14]. *Lgd1* is not an essential gene, as homozygous mutant mice are fertile and of normal appearance [15]. Nevertheless, a recent report shows that it is required for nuclear envelope formation after mitosis [16]. In mice, the loss of function of *Lgd2* leads to perinatal death due to a failure of breathing. The failure is a consequence of a brain defect [15, 17, 18]. Loss of *LGD2* function in humans results in autism and mental retardation and increases NF-κB activity [19]. It has also been implicated in Toll and PKC signalling [17, 20].

The interaction of Lgd with Shrub is mediated by the DM14 domains 1 and 3 (DM14-1 and DM14-3), which function in a redundant manner [6, 8]. The crystallisation of DM14-3 alone and in complex with a truncated version of Shrub revealed (1) that the DM14 domains are helical hairpins and (2) that the odd-numbered DM14 domains bind via a positively charged surface to the negative surface of Shrub, which is also required for its homo-polymerisation at the LM [6]. Hence, polymerisation of Shrub on the LM of the ME and binding of Shrub to Lgd is mutually exclusive. This mutual exclusivity suggests an antagonistic functional relationship between the two proteins, which has been also supported by cell culture experiments [5]. In contrast, genetic interaction experiments in *Drosophila* suggest that Lgd might be required for the activity of Shrub in vivo [8]. Despite this genetic evidence for a positive relationship, the loss of function phenotypes of *lgd* and *shrub* differ significantly: While they have in common the ectopic ligand-independent activation of the Notch signalling pathway and cell over-proliferation, *shrub* mutants display the additional loss of apicobasal polarity of the imaginal disc epithelium, which is not observed in *lgd* mutants [7, 8]. The reason for the phenotypic difference, neoplastic in *shrub* and hyperplastic in *lgd* mutants, is not understood.

The Notch signalling pathway consists of three core components, a ligand of the DSL family of transmembrane proteins, the Notch receptor and a transcription factor of the CSL family (reviewed in [21]). The binding of a ligand of the DSL family to Notch elicits two consecutive proteolytic cleavages that release the ICD of Notch (NICD) into the cytosol from which it travels into the nucleus. In the nucleus, it associates with a CSL transcription factor (Suppressor of Hairless (Su(H)) in *Drosophila*) to activate target gene expression. The ligand-dependent first cleavage of Notch is performed by the metalloprotease ADAM10 and results in shedding of its extracellular domain (ecto-domain shedding). The truncated intermediate, termed NEXT (*N*otch *ex*tracellular *t*runcation) is then liberated from the membrane by intra-membrane cleavage by the γ-secretase complex. The cleavage by γ-secretase is relatively unspecific and appears to occur if the ECD of a TMP is below 100 amino acids [22].

Notch at the plasma membrane is constantly turned over independently of signalling by the interplay of exo- and endocytosis (reviewed in [10]). Following endocytosis, Notch is transported through the endosomal pathway to be degraded in the lysosome. On its journey to the lysosome, Notch is incorporated into ILVs of MEs to ensure complete degradation. In ESCRT mutants, where ILV formation is compromised, Notch appears to remain in the LM of the ME. According to the current model explaining the ectopic activation of the Notch pathway in ESCRT mutants, only the ECD of Notch, which protrudes into the lumen, is degraded in late MEs and lysosomes due to the activity of the acidic hydrolases. The degradation of the ECD creates a NEXT-like intermediate in the LM that is cleaved by γ-secretase to release NICD. The NICD created by this alternative ecto-domain shedding travels into the nucleus and activates target gene expression. A similar model has been proposed for the activation of Notch in *lgd* mutants [7]. However, although plausible, the models have neither been tested nor proven. Recent evidence suggests that a fraction of Notch indeed remains at the LM of the ME in *lgd* mutant cells [23]. However, it is not known whether this fraction is activated. Moreover, in contrast to *shrub* mutants, ILV formation takes place in *lgd* mutant cells [8]. Thus, it is not clear why Notch remains at the LM in *lgd* mutant MEs.

Two E3 ubiquitin ligases, termed Suppressor of deltex (Su(dx)) and Deltex (Dx), regulate the endocytosis of Notch and also its subsequent incorporation into ILVs [24]. Over-expression of Su(dx) promotes Clathrin-independent endocytosis of Notch and its subsequent incorporation into ILVs [24, 25]. Moreover, loss of its function results in a similar, albeit much weaker ectopic activation of Notch, as has been observed for *lgd* mutants [26]. The qualitative similarity of the phenotypes raises the possibility that both genes act within the same pathway. The activity of Dx promotes Clathrin-mediated endocytosis of Notch and antagonises its incorporation into ILVs by Su(dx) on the LM of the ME.

Here, we show that loss of function of *lgd* constitutes a state of reduced activity of Shrub. This reduction of activity seems to disturb the function of the ESCRT machinery and therefore causes a slight decrease in the rate of ILV formation. This defect probably causes the escape of a fraction of endosomal Notch from incorporation into ILVs and this fraction is activated. We show that LGD1 (in *Drosophila* Lgd) binding does not affect the conformation of CHMP4B (in *Drosophila* Shrub) in solution, suggesting that LGD1/Lgd is not a chaperone of CHMP4B/Shrub. In addition, we show that Su(dx) and Lgd act in separate pathways that both regulate the amount of Notch that remains at the LM of the ME during endosomal trafficking.

## Results

### Lgd is required for efficient incorporation of Notch into ILVs

**Fig. 1:**
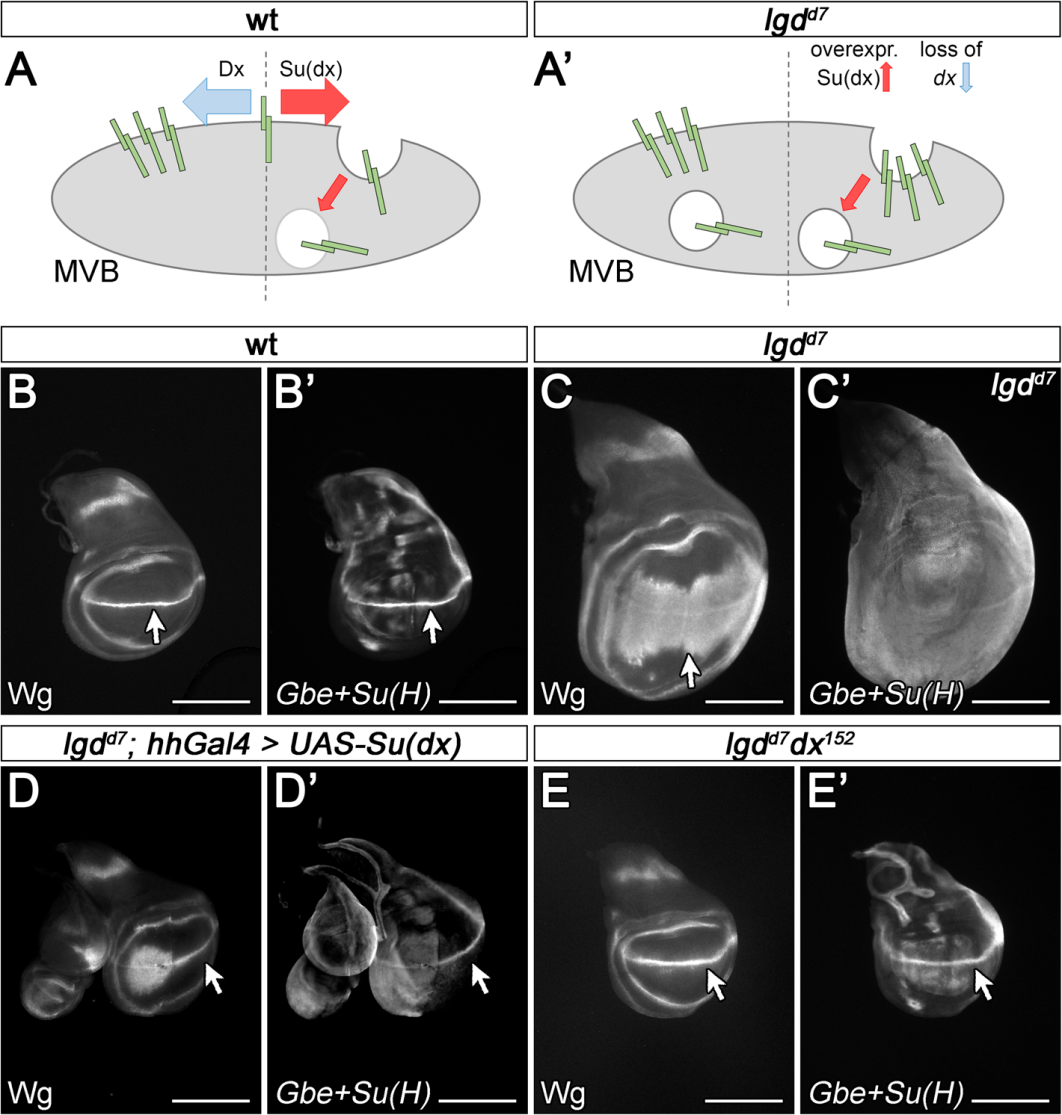
A fraction of Notch remains at the LM of *lgd* mutant MEs and is activated. (**A**, **A’**) Dx and Su(dx) act antagonistically to determine the fraction of Notch remaining at the LM of the ME. Over-expression of Su(dx) or loss of *dx* function favours incorporation of Notch into ILVs. (**B**–**C’**) Expression of the Notch target Wg and the Notch activity reporter *Gbe+Su(H)* in wild-type and *lgd* mutant discs. The expression domain of Wg along the D/V boundary of the wing anlage (arrow in **B**) is dramatically expanded in *lgd* mutant discs (arrow in **C**). In addition, the complex expression pattern of *Gbe+Su(H)* seen in wild-type is replaced by a uniform staining in the mutant discs (**B’**, **C’**). (**D**, **D’**) Over-expression of Su(dx) with *hh*Gal4 in the posterior compartment normalises the expression of both read-outs of Notch activity (arrow). A similar effect is seen if the function of *dx* is removed in *lgd* mutant discs (**E**, **E’**, arrow). Note that the normal expression of the Notch targets, e.g. along the D/V boundary (arrow in **D**–**E’**), is not affected by the manipulations. Scale bars (**B**–**E’**) 200 μm. At least ten wing imaginal discs were analysed for each genotype

Elegant experiments with a Notch variant simultaneously tagged with GFP and mCherry in its ICD suggest that a fraction of Notch remains at the LM of the ME in *lgd* mutant cells [23]. We reasoned that if this is true and if this fraction is responsible for the ligand-independent ectopic activation of the pathway, the activation should be suppressed if Notch is forced to be incorporated into ILVs. This can be achieved by over-expressing Su(dx), or loss of function of its antagonist *dx* [24] (Fig. 1A, A’). *lgd* mutant wing imaginal discs are over-proliferated and display an expansion of the Notch target gene *wg* in the wing area from the dorso-ventral compartment boundary (D/V boundary) into the wing anlage (Fig. 1B, C). In addition, the more sensitive activity reporter *Gbe+Su(H)*, normally expressed in a complex pattern, is uniformly expressed in all disc cells (Fig. 1B’, C’).

We found that the over-expression of Su(dx) in *lgd* mutant discs resulted in the normalisation of the expression of both activity reporters, indicating that the ectopic ligand-independent activation of the Notch pathway is suppressed (Fig. 1D, D’, arrow compare with B–C’). A similar suppression of the ectopic activity of the Notch pathway was observed if the function of *dx* was lost in *lgd* mutant discs (Fig. 1E, E’). Although the loss of *dx* function also results in less efficient endocytosis of Notch, this effect is weak, as well as its phenotypic consequences [27]. Therefore, we think that it alone cannot account for the nearly complete normalisation of Notch signalling observed in the *dx lgd* double mutants. Note that in both experiments the expression of *Gbe+Su(H)* and Wg in their regular expression domains, e.g. along the dorso-ventral compartment boundary of the wing disc, was unaffected (Fig. 1D–E’, arrow compare with B and B’). These domains are induced by ligand-dependent Notch signalling. Hence, only the ectopic, ligand-independent activity of Notch is suppressed by the manipulations. This ectopic activity is caused by the endosomal defect of *lgd* mutants [14]. Collectively, the results confirm the notion that in *lgd* mutants a fraction of Notch remains at the LM of the ME and indicate that this fraction is ectopically activated.

### Su(dx) and Lgd do not operate in a linear pathway

Similar to *lgd*, loss of function of *Su(dx)* leads to the ectopic activation of the Notch pathway and is involved in the ESCRT-mediated incorporation of Notch into ILVs [24]. Although the ectopic activation is much weaker than that observed in *lgd* mutants and its severity is dependent on temperature, the qualitative similarity of the phenotype of *lgd* and *Su(dx)* mutants raised the possibility that the two genes act within the same pathway. *Su(dx)* mutants are viable and fertile at any temperature and display an ectopic activation of Notch at 29 °C [26]. We here found that, in contrast to *lgd* mutants, this activation is too weak to be detected even with the sensitive *Gbe+Su(H)*, even at 29 °C (see clonal analysis in Additional file 1: Figure S1A-D”’). If both genes act within the same linear pathway, the double mutant should display the stronger *lgd* mutant phenotype. However, *lgd Su(dx)* double mutants displayed a phenotype different from that of each single mutant (Additional file 2: Figure S2). At 29 °C where the function of *Su(dx)* is most required, the double mutants died during the early phase of the third larval instar stage (L3), which is earlier than *lgd* single mutants (Additional file 2: Figure S2A). At 25 °C, the flies developed further, but did not pupariate, which is still an earlier time of death than *lgd* mutants (Additional file 2: Figure S2A). At 18 °C, where the function of *Su(dx)* is least required, many flies reached the early pupal stage, just like *lgd* mutants (Additional file 2: Figure S2A).

The phenotype of the *lgd Su(dx)* wing discs varied correspondingly: At 18 °C, the discs were similar to *lgd* mutant discs and Wg and *Gbe+Su(H)* were ectopically expressed (Additional file 2: Figure S2B, B’). However, the ectopic activation of Notch normally observed in *lgd* mutants is suppressed in the dorsal compartment (Additional file 2: Figure S2B, B’ arrow). This suggests that the function of Su(dx) is weakly active also at cold temperatures. At 25 °C, the discs were only slightly over-proliferated, Wg was only faintly detectable and *Gbe+Su(H)* expressed uniformly (Additional file 2: Figure S2C, C’). Overall, the discs were smaller and the wing area was not flattened out as in *lgd* mutant discs, but more irregularly folded. The discs also displayed increased cell death. At 29 °C, the double mutant discs were too small for a proper analysis.

Altogether, the results indicate that *lgd Su(dx)* double mutants display a phenotype that is different from that of the single mutants. In the experiments, a null allele for *lgd* was used, while the Su(dx) allele is a strong hypomorph [28]. The observed synergistic phenotype of the *Su(dx) lgd* double mutant therefore suggests that the two genes operate either in independent processes or in a common process that is not linear. Consistent with this conclusion, previous work suggests that the two genes act independently on the ESCRT machinery, Su(dx), via mono-ubiquitylation, enables the recognition of Notch by ESCRT-0, whereas Lgd acts on the ESCRT-III core component Shrub/CHMP4 [7, 29].

### Analysis of *lgd* mutant MEs suggests a decrease in the rate of ILV formation

**Fig. 2:**
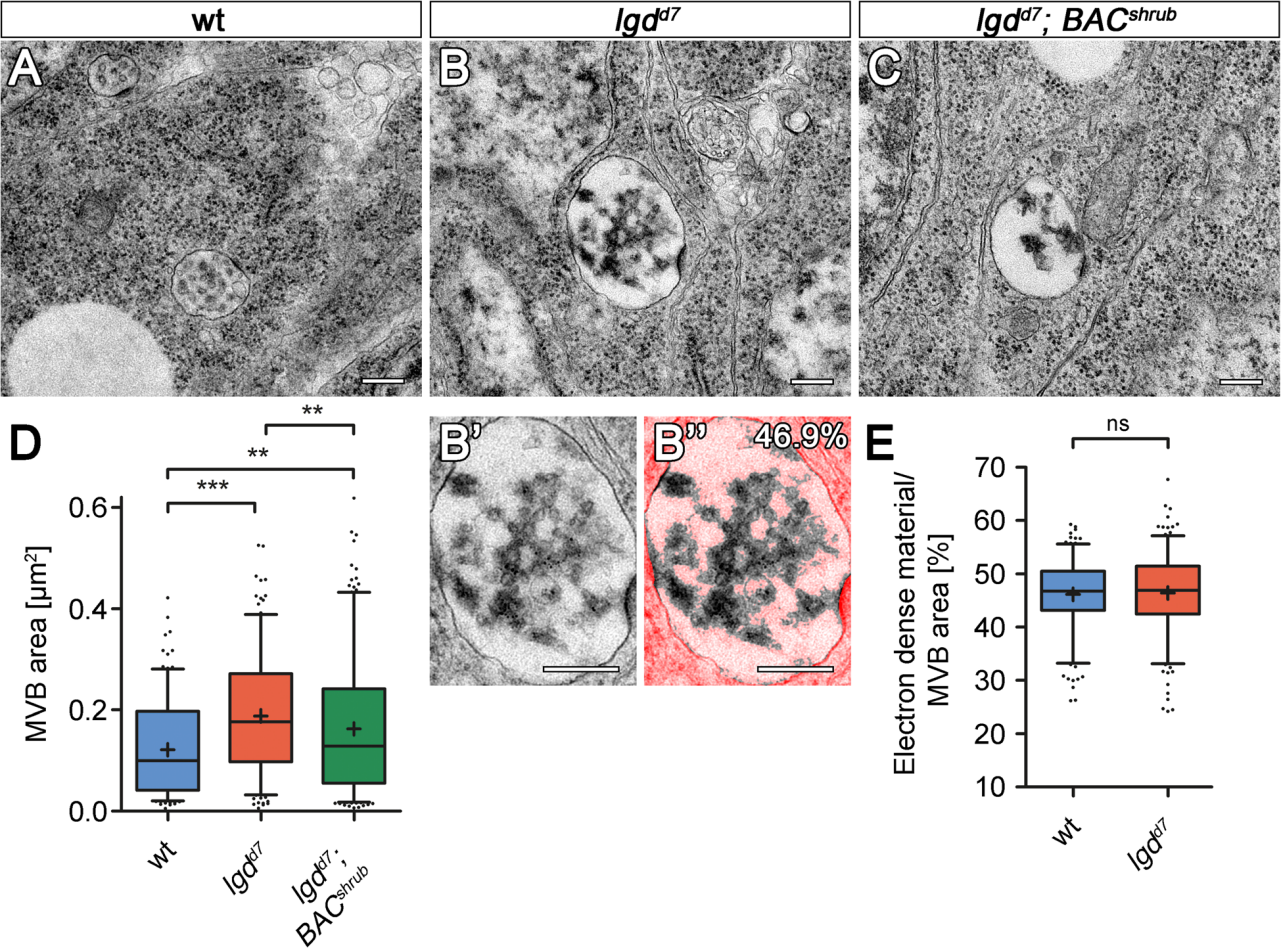
*lgd* mutant MEs have a normal ILV content. (**A**–**E**) Ultra-structural analysis of *lgd* mutant and *lgd* cells rescued by additional genomic copies of *shrub* (*lgd*^*d7*^; *BAC*^*shrub*^). Representative TEM pictures of MVBs of wild-type (**A**), *lgd*^*d7*^ mutant (**B**) and rescued *lgd*^*d7*^ mutant (**C**) wing disc cells. (**B”**) As an example, colourized overlay of determined ILV area (46.9% of total MVB area) with the original electron microscopy image of the MVB is shown in (**B’**). Highlighted in bright red is the area which is not considered as ILV content/electron-dense material within the MVB. (**D**) Statistical analysis of MVBs of wild-type (blue), *lgd* mutant (red) and rescued *lgd* mutant (green) cells. The average area of *lgd* mutant MVBs is significantly increased in comparison to MVBs of wild-type cells. Furthermore, the enlargement of endosomes in *lgd* mutants is partially rescued in *lgd* mutant cells expressing two additional copies of *BAC-shrub*. (**E**) Pixelwise quantification of electron-dense material per area within the lumen of wt (blue) and *lgd*^*d7*^ mutant (red) MVBs to measure ILV formation (as shown in **B’**, **B”**). A self-written macro for the image processing software “Fiji” was used (see the “Methods” section and [30]). Area quotients of several MVBs are collected and blotted in a box blot. The ILV content in *lgd*^*d7*^ mutant is not changed in comparison to the wild-type [(**D**, **E**) wt *n* = 212 MVBs; *lgd*^*d7*^ *n* = 230 MVBs; *lgd*^*d7*^; *BAC*^*shrub*^ *n* = 277; (**D**) Kruskal-Wallis test, Dunn’s multiple comparison test *p* < 0.001 (***), *p* < 0.01 (**); (**E**) Mann-Whitney test, two-tailed; box plots: whiskers: 5–95 percentile; mean shown as “+”]. Scale bars (**A**–**C**) 250 nm

The remaining of Notch at the LM of the ME of *lgd* mutants can be caused by either a failure or reduction of ILV formation, or a failure to incorporate Notch into ILVs. Previous EM analysis revealed that ILV formation takes place in *lgd* mutants [8]. To explore the possibility that ILV formation is reduced, which would also allow a fraction of Notch to remain at the LM, we compared the electron-dense content of wild-type (wt) and *lgd* mutant MEs. To do so, we measured pixelwise the electron density in the lumen of MEs captured with the TEM as MVBs (Fig. 2A–B’, D, E, see M&M and [30] for further details).

We first tested the suitability of the assay by several means. Firstly, we determined the electron-dense content of MEs of cells where N-terminal Myc-tagged Shrub or ShrubΔauto was expressed with help of the Gal4/Gal80^ts^ system for 13 h (Additional file 3: Figure S3). Myc-ShrubΔauto lacks the auto-inhibitory C-terminus and probably accumulates on the LM of the ME because it lacks the MIM interaction sites required for the disassembly of the ESCRT-III polymer by Vps4 [31]. In that way, it is thought to abolish the function of ESCRT-III over time, as the complex cannot be disassembled. In line with this notion, we found that, in contrast to the expression of Myc-Shrub, the expression of Myc-ShrubΔauto leads to an enlargement of the MEs, as well as a significant decrease in the luminal electron-dense content (Additional file 3: Figure S3C-F). These results show also for *Drosophila* that inactivation of ESCRT-III, in this case due to impaired Vps4 recruitment, results in a failure of ILV formation and demonstrate the suitability of our assay.

**Fig. 3:**
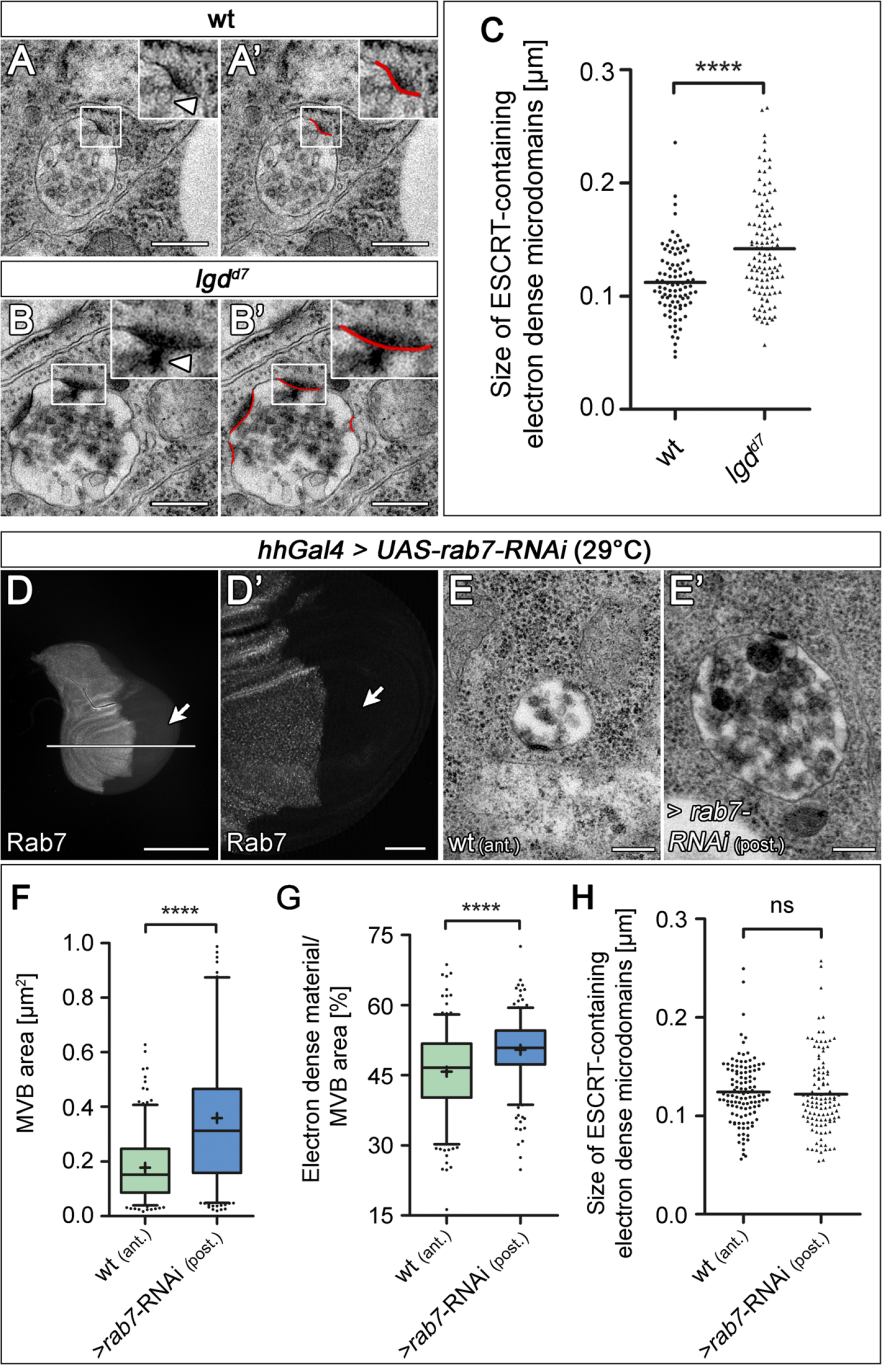
The size of ESCRT-containing electron-dense microdomains is enlarged in *lgd* mutant MEs. Representative TEM pictures of MVBs of wild-type (**A**, **A’**) and *lgd*^*d7*^ mutant (**B**, **B’**) wing disc cells. (**A’**, **B’**) Defined and measured ESCRT-containing electron-dense microdomains of the MVBs shown in **A** and **B** are highlighted in red. Magnification of ESCRT-containing electron-dense microdomains where the associated, nascent ILV is clearly observable (arrowhead). The overall morphology of the MVBs in *lgd*^*d7*^ mutants appears to be normal (**B**, **B’**). (**C**) Statistical analysis of the ESCRT-containing electron-dense microdomains of wild-type and *lgd*^*d7*^ mutant MVBs in wing imaginal disc cells. The average size of *lgd* mutant electron-dense microdomains is significantly increased in comparison to the wild-type [wt *n* = 72 MVBs (90 ESCRT-containing electron-dense microdomains); *lgd*^*d7*^ *n* = 87 MVBs (111 ESCRT-containing electron-dense microdomains); Mann-Whitney test, two-tailed *p* < 0.0001 (****); scatter dot plot, mean shown as a “line”]. (**D**–**H**) Analysis of MEs of cells that are depleted of Rab7 function. (**D**, **D’**) A wing disc depleted of Rab7 function in the posterior compartment by the continuous expression of *rab7*-RNAi with *hh*Gal4 (arrow). The magnification in (**D’**) highlights the efficiency of the depletion in the posterior compartment (arrow). (**E**, **E’**) Representative MEs/MVBs of anterior wild-type and posterior depleted cells. Analysis of the size (**F**), luminal content (**G**) and size (**H**) of the ESCRT-containing electron-dense microdomains of the wild-type and Rab7-depleted MEs/MVBs. While the average size and the luminal content is increased, there is no change in the size of the ESCRT-containing electron-dense microdomains in comparison to wild-type [(**F**, **G**) wt (ant.) *n* = 253 MVBs; (**F**) *rab7-*RNAi (post.) *n* = 282 MVBs; (**G**) *rab7-*RNAi (post.) *n* = 280 MVBs; (**F**, **G**) Mann-Whitney test, two-tailed *p* < 0.0001 (****); box plots: whiskers: 5–95 percentile; mean shown as “+”. (**F**) Values greater than 1 μm^2^ are not shown (*rab7*-RNAi (post.): 8 out of 282 MVBs). (**H**) wt (ant.) *n* = 86 MVBs (114 ESCRT-containing electron-dense microdomains); *rab7*-RNAi (post.) *n* = 92 MVBs (110 ESCRT-containing electron-dense microdomains) Mann-Whitney test, two-tailed; scatter dot plot; mean shown as a “line”]. Scale bars (**A**–**B’**, **E**, **E’**) 250 nm; (**D**) 200 μm; (**D’**) 50 μm. (**D**, **D’**) At least ten wing imaginal discs were analysed for each genotype

Previous work suggests that the lifetime of the endosome is increased in *lgd* mutant cells [7]. To investigate what effect an increase of lifetime of the endosome might have on the number of ILVs in MEs, we analysed the MEs of *rab7*-depleted cells. Rab7 organises the fusion of the ME with the lysosome, which terminates its existence [10]. Consequently, loss of *rab7* function increases the lifetime of the ME. We have previously shown that ILV formation occurs and the endosomes mature in all aspects tested in *rab7*-depleted cells [32]. We here found that the MEs of *rab7*-depleted disc cells were increased in size and had a higher electron density compared to MEs of wild-type cells (Fig. 3F, G) [32]. This finding suggests that the increase in lifetime allows more rounds of ILV formations to occur, which results in a higher number of ILVs in MEs. They are also consistent with previous work demonstrating that *rab7*-depleted MEs are enlarged and contain more ILVs in HeLa cells [33]. However, *lgd* mutant MEs, although significantly enlarged, had a similar electron density in their lumen to wild-type MEs (Fig. 2A–E). Hence, the number of ILVs per area is the same in wild-type and *lgd* mutant MVBs and not increased as expected for MEs with increased lifetime. Our analysis therefore suggests that the rate of ILV formation is slightly reduced for *lgd* mutant MEs.

### The size of ESCRT-containing electron-dense microdomains is increased in *lgd* mutant MEs

During our TEM analysis, we regularly observed electron-dense sites at the LM, which were often invaginated and sometimes even associated with a nascent ILV (Fig. 3A–B’, arrow in 3A, B). These sites have been observed in various cell types and constitute Clathrin-coated assemblies of the ESCRT machinery (here termed ESCRT-containing electron-dense microdomains) [34–38]. The coat is recruited by Hrs of ESCRT-0 [35, 39]. Consistently, a reduction in the activity of the ortholog of Shrub causes an increased accumulation of ESCRT-0 and Clathrin in *C. elegans* [35, 40, 41]. Since Lgd interacts with Shrub, we measured whether the size of the ESCRT-containing electron-dense microdomains is changed in *lgd* mutant disc cells. We found a highly significant increase of the average length of these microdomains of *lgd* mutant compared to wild-type MEs (Fig. 3C).

The increase of the ESCRT-containing electron-dense microdomains might be a non-specific scaling effect of the size increase of *lgd* mutant MEs (Fig. 2D), or a specific effect that indicates a disturbance of ESCRT function. To discriminate between these possibilities, we measured the size of the ESCRT-containing electron-dense microdomains in *rab7*-depleted cells. We found that the length of these microdomains was not increased. This finding indicates that the phenotype of the two mutations that causes an increase in size of the ME is different in respect to the ESCRT-containing electron-dense microdomains, suggesting that the size increase of the microdomains is a specific effect of the loss of *lgd* function (Fig. 3H). The increase suggests a change in the activity of the later acting part of the ESCRT machinery (after ESCRT-0) in the absence of the function of *lgd*.

### A reduction of *shrub* activity causes a phenotype similar to that of *lgd* mutants

**Fig. 4:**
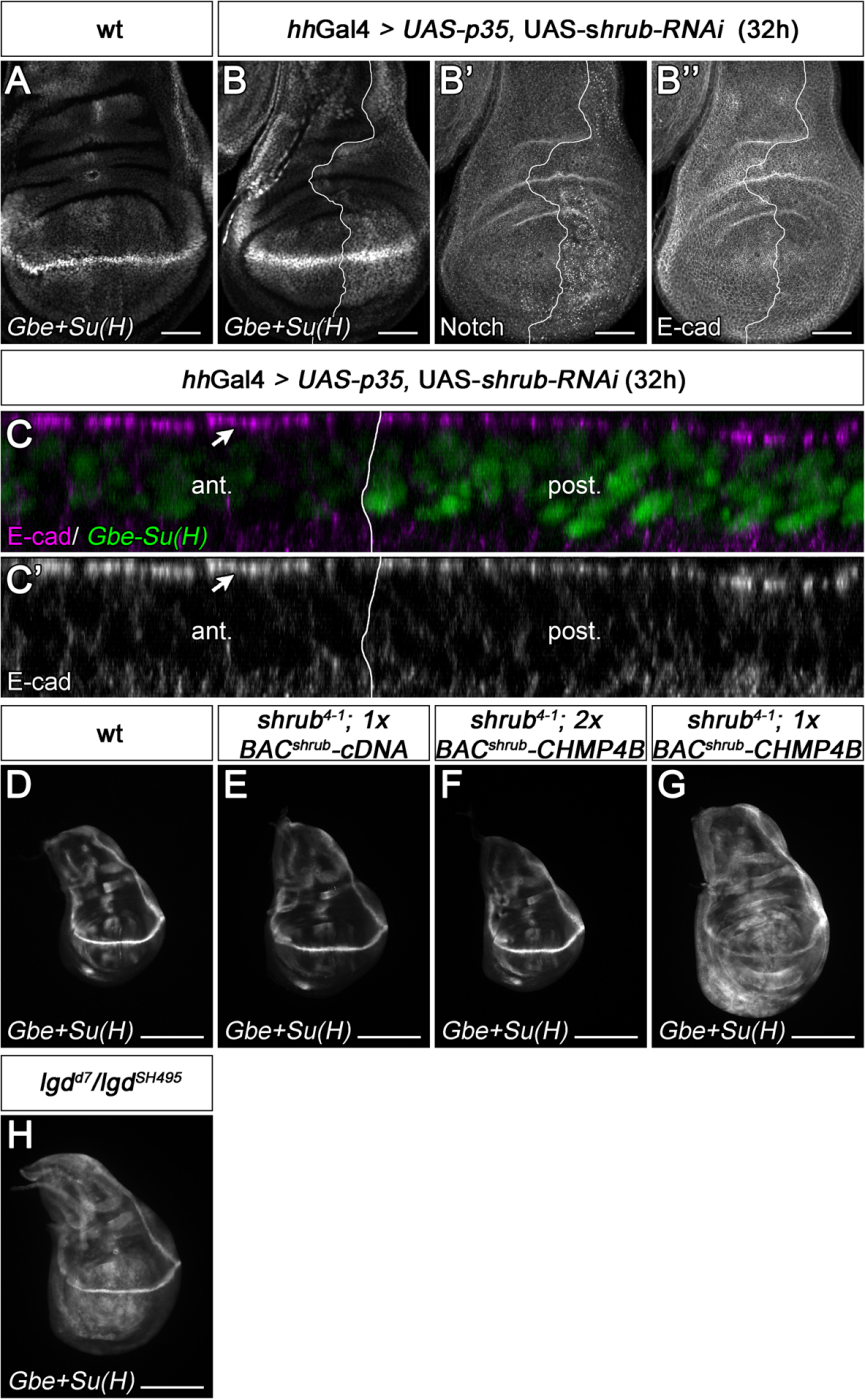
Reduction of *shrub* function causes a phenotype that resembles that of *lgd* mutants. (**A**) Expression of *Gbe+Su(H)* in a control wild-type disc. (**B**–**C’**) Depletion of *shrub* function by expression of UAS *shrub-RNAi* with *hh*Gal4 *tub*Gal80^ts^ for 32 h (in the posterior compartment). The expression of *Gbe+Su(H)* is significantly increased in the depleted posterior area (**B**, **C**). The depleted cells accumulate Notch in enlarged endosomes (**B’**), but the expression of E-cad is not affected (**B”**). (**C**, **C’**) Z-stack of the disc shown in (**B**–**B”**) to show the apical-basal axis of the disc cells. The comparison of the cells of the anterior with cells of the posterior compartment reveals that the subcellular localisation of E-cad is not affected by the depletion of *shrub*. (**D**–**H**) Rescue of *shrub* null mutants by *BAC*^*shrub*^*-CHMP4B*. (**D**) Expression of *Gbe+Su(H)* in a control wild-type disc. (**E**) A *shrub* mutant disc completely rescued by one copy of the control *BAC*^*shrub*^*-cDNA* construct. (**F**) A similar rescue is observed with two copies of *BAC*^*shrub*^*-CHMP4B*. (**G**) One copy of *BAC*^*shrub*^*-CHMP4B* leads only to a partial rescue. *Gbe+Su(H)* is slightly ectopically expressed. It resembles the expression of *Gbe+Su(H)* in a hypomorphic situation of *lgd* shown in (**H**). Scale bars (**A**–**B”**) 50 μm; (**D**–**H**) 200 μm. At least ten wing imaginal discs were analysed for each genotype

A reduction of the activity of the later acting ESCRT machinery, more precise of Shrub, could also explain the differences in the phenotype of *shrub* and *lgd* mutants: the reduction of its activity might result in a hyperplastic phenotype caused by the ectopic activation of the Notch pathway (*lgd* mutant phenotype), while complete loss of its activity causes a neoplastic phenotype that includes the loss of epithelial polarity in addition to the other traits (*shrub* mutant phenotype). To evaluate this assumption, we sought to create a hypomorphic situation of *shrub*. To do so, we temporarily expressed an efficient *shrub*-RNAi construct. This temporary depletion was achieved by using *hh*Gal4 in combination with the temperature-sensitive form of its suppressor Gal80 (Gal80^ts^) to drive the expression of *shrub*-RNAi (*hh*Gal4 *tub*Gal80^ts^ *UAS shrub*-RNAi). The experimental set up causes depletion of *shrub* only in the posterior half (compartment) of the wing disc for a chosen time interval [30]. With this approach, we observed ectopic activation of the Notch activity reporter *Gbe+Su(H)* already after 32 h of *shrub*-RNAi expression (Fig. 4A–C). The disc cells showed no defect in epithelial polarity, judged by the normal localisation of the apically located Notch and the adherens junction marker E-cadherin (E-cad) (Fig. 4B”–C’). This result suggests that reduction of *shrub* activity causes ectopic activation of the Notch pathway without loss of epithelial polarity, as it can be observed in *lgd* mutants.

In a complementary experiment, we asked to what extent Shrub can be replaced by its mammalian ortholog CHMP4B. To achieve physiological expression of CHMP4B, we used a BAC that contains the genomic *shrub* locus (*BAC*^*shrub*^). We replaced the *shrub* transcription unit of *BAC*^*shrub*^ either by a cDNA of CHMP4B (*BAC*^*shrub*^*-CHMP4B*) or, as a control, by a cDNA of *shrub* (*BAC*^*shrub*^*-cDNA*). *shrub* null mutant flies die during embryogenesis [42]. We found that one copy of *BAC*^*shrub*^ or *BAC*^*shrub*^*-cDNA* rescued the *shrub* mutant phenotype completely (Fig. 4D, E and Additional file 4: Figure S4A). The rescued flies looked normal and were fertile. In contrast, a *BAC*^*shrub*^ variant in which three inactivating charge reversal mutations of the acidic interaction surface of Shrub were introduced (*BAC*^*shrub*^*-*mut2 mutation of E86R, E90R, E93R [4, 5]) failed to rescue (Additional file 4: Figure S4A). This indicates that the transcription unit of *shrub* is indeed responsible for the rescue of the BACs.

Two copies of BAC^shrub^-*CHMP4B* rescued *shrub* mutants, although the resulting adult flies were sterile (both sexes) (Additional file 4: Figure S4B). However, one copy of BAC^*shrub*^*-CHMP4B* only partially rescued *shrub* mutants to the pupal stage, indicating that it cannot provide sufficient *shrub* activity to complete development (Additional file 4: Figure S4B). It therefore constitutes another more stable hypomorphic *shrub* mutant situation that we analysed.

**Fig. 5:**
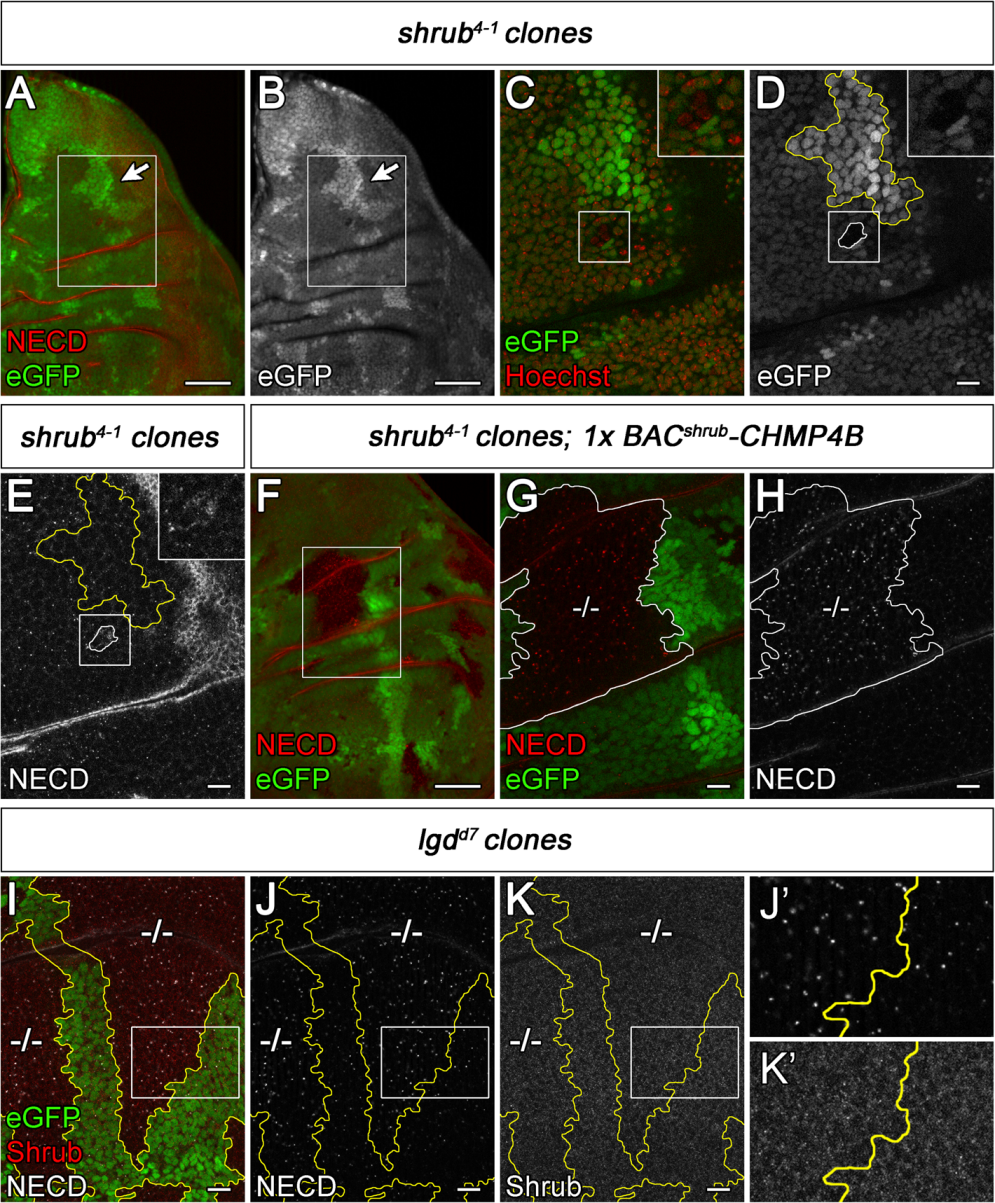
Comparison of *shrub clones* in the absence or presence of one copy of *BAC*^*shrub*^*-CHMP4B* in the genome with *lgd*^*d7*^ mutant cell clones. Clones are labelled by the absence of GFP. (**A**–**E**) *shrub* mutant cell clones are rare and comprise only a few cells. (**A**, **B**) Overview of the notal region of a wing disc bearing a *shrub* mutant clone. (**C**–**E**) Magnification of the region boxed in (**A**, **B**). A small *shrub* mutant clone is boxed and shown at higher magnification in the insert. The few surviving mutant cells contain enlarged Notch-positive MEs (**E**). In contrast, large orphan wild-type twin-clones (GFP-positive (arrow in **A**, **B** and outlined in yellow **D**, **E**) are present in these discs. (**F**–**H**) *shrub* mutant cell clones partially rescued with one copy of *BAC*^*shrub*^*-CHMP4B.* (**F**) Several large mutant clones can be observed, indicating that the lethality is suppressed. (**G**, **H**) Magnification of the region boxed in (**F**) which includes a large clone. The partially rescued cells of the clone display a similar endosomal defect as the cells of *lgd* mutant clones, shown in (**I**–**K**). In both cases, the enlarged MEs of the mutant cells accumulate Notch. (**K**, **K’**) Note that the levels of Shrub in the *lgd* mutant cells are similar to that of the neighbouring wild-type cells. Scale bars (**A**, **B**, **F**) 50 μm, (**C**–**E**, **G**–**K**) 10 μm. At least ten wing imaginal discs were analysed for each genotype

While Notch target gene expression was comparable to wild-type in flies rescued to adulthood by a copy of *BAC*^*shrub*^*-cDNA* or two copies of *BAC*^*shrub*^*-CHMP4B* (Fig. 4D–F, Additional file 4: Figure S4C, C’), a slight ectopic activation of them was observed in the *shrub* mutants partially rescued by one copy of *BAC*^*shrub*^*-CHMP4B* to an extent also observed in weak *lgd* mutants, e.g. as *lgd*^*SH495*^/*lgd*^*d7*^ (Fig. 4G, H). In addition, the discs were slightly enlarged as in the case of weak *lgd* mutants (Fig. 4G, H, compare with D). Again, the apicobasal polarity of the disc epithelium was unaffected, indicated by the normal localization of the basolateral marker Discs large (Dlg), the adherens junction marker E-cad and apically located Crumbs (Crb) (Additional file 4: Figure S4G-J). To further determine whether the partially rescued *shrub* mutant cells had a similar phenotype as *lgd* mutant cells, we turned to clonal analysis. *shrub* mutant cell clones are rare and small, indicating that loss of *shrub* function is cell lethal (Fig. 5A–E). In contrast, the mutant clones were large and frequent if one copy of *BAC*^*shrub*^*-CHMP4B* was present in the genome (Fig. 5F–H). The cells in the *shrub* mutant cell clones partially rescued with one copy of BAC^*shrub*^*-CHMP4B* displayed an increased number of Notch-positive MEs (Fig. 5F–H). This phenotype resembled that seen in *lgd* mutant cells (Fig. 5I–K’) [13, 14].

**Fig. 6:**
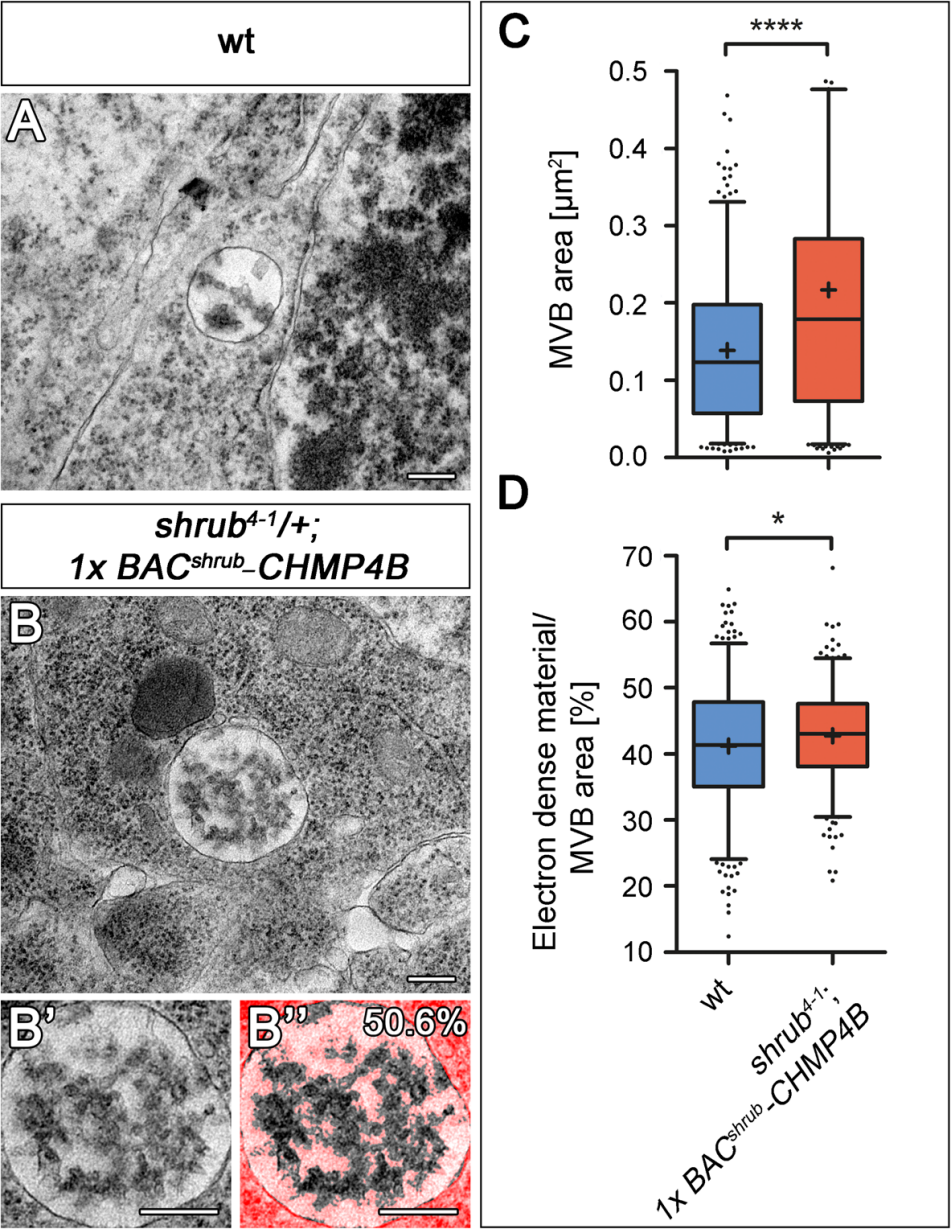
Reduction of *shrub* activity causes moderate enlargement of MVBs. (**A**–**D**) Ultra-structural analysis of *shrub* mutant cells partially rescued by *BAC*^*shrub*^*-CHMP4B* (*shrub*^*4-1*^; *BAC*^*shrub*^*-CHMP4B/+*). Representative TEM pictures of MVBs of wild-type (**A**) and partially rescued *shrub* mutant (**B**) wing disc cells. (**B”**) Colourized overlay of estimated ILV area (50.6% of total MVB area) with the original EM image of the MVB shown in (**B’**). The area that is not considered as ILV content/electron-dense material within the MVB is highlighted in bright red. (**C**) Statistical analysis of the MVBs of wild-type (blue) and partially rescued *shrub* mutant (red) cells. The average area of MVBs in *shrub* mutant cells is significantly increased. (**D**) Quantification of electron-dense material per area within the lumen of wt (blue) and partially rescued (red) MVBs to measure ILV formation (as shown in **B’**). There is no change in the ILV content in the partially rescued *shrub* mutant cells in comparison to wild-type [(**C**, **D**) wt *n* = 337 MVBs; (**C**) *shrub*^*4-1*^*; BAC*^*shrub*^*-CHMP4B/+ n* = 271; (**D**) *shrub*^*4-1*^*; BAC*^*shrub*^*-CHMP4B/+ n* = 263; (**C**) Mann-Whitney test, two-tailed *p* < 0.0001 (****). (**C**) Values greater than 0.5 μm^2^ are not shown (wt, 1 out 337 MVBs; *shrub*^*4-1*^*; BAC*^*shrub*^*-CHMP4B/+*, 10 out of 271 MVBs). (**D**) Unpaired *t* test, *p* < 0.05 (*) (box plot: whiskers: 5–95 percentile; mean shown as “+”)]. Scale bar (**A**, **B**, **B’**) 250 nm

The TEM analysis of wing disc cells revealed that, as in *lgd* mutants, the MEs of the partially rescued *shrub* mutant cells (*shrub*^*4-1*^*/+; 1x BAC*^*shrub*^*-CHMP4B*) contained ILVs and were enlarged (Fig. 6A–C). Moreover, as in the case of *lgd* mutants, the luminal electron-dense content of MEs of the partially rescued mutants was similar to that of wild-type MEs (Fig. 6D). Thus, also at the ultra-structural level, the phenotype of the partially rescued *shrub* mutants resembled that of *lgd* mutants.

The ectopic activation of Notch target genes seen in the *shrub* mutants partially rescued by one copy of *BAC*^*shrub*^*-CHMP4B* was strongly enhanced by the loss of one functional copy of *lgd* (genotype *shrub*^*4-1*^*, lgd*^*d7*^/ *shrub*^*4-1*^*, +*; *BAC*^*shrub*^*-CHMP4B*/+). The phenotype closely resembled that of complete loss of *lgd* function (Additional file 4: Figure S4F, F’, compare with D, D’ and Fig. 1C, C’). Hence, just like Shrub, CHMP4B appears to interact with Lgd in vivo and this interaction is required for its function. Altogether, the results indicate that a reduction of the activity of *shrub* causes a phenotype that is very similar to that of loss of *lgd* function. Moreover, they indicate that proteins of the Lgd family enhance the activity of CHMP4 proteins. If their function is lost, the activity of CHMP4 proteins is reduced, but not abolished.

### Elevating the level of Shrub rescues the *lgd* mutant phenotype

**Fig. 7:**
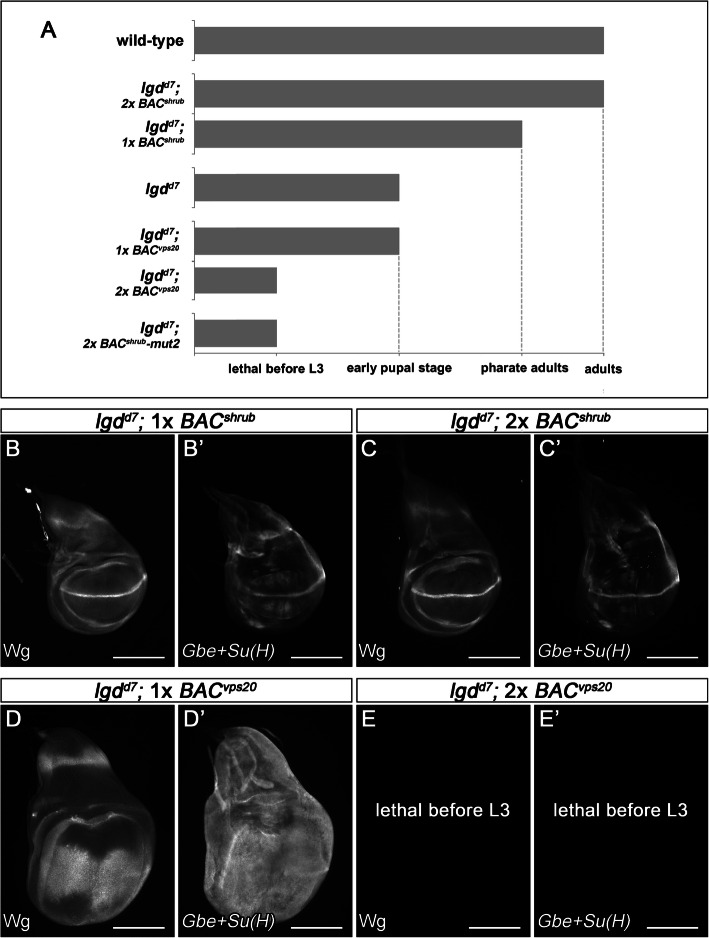
Rescue of *lgd* mutants by extra copies of *BAC*^*shrub*^ or *BAC*^*vps20*^*.* (**A**) Time of death of *lgd* mutants rescued with one or two copies of *Bac*^*shrub*^, *Bac*^*shrub*^*-mut2* or *Bac*^*vps20*^. (**B**–**C’**) The addition of one or two copies of *BAC*^*shrub*^ results in the normalisation of the expression patterns of Wg and *Gbe+Su(H)* (compare with Fig. 1B–C’). (**D**, **D’**) The addition of one copy of *BAC*^*vps20*^ does not modify the *lgd* mutant phenotype (compare with Fig. 1C, C’), while the presence of two extra copies results in premature death during development (**A**, **E**, **E’**). At least ten wing imaginal discs were analysed for each genotype

If loss of *lgd* function constitutes a state of reduced *shrub* activity, it might be possible to compensate for this reduction by adding extra genomic copies of *shrub*. To do so, we introduced varying numbers of *BAC*^*shrub*^ into the genome of *lgd* null mutants. We found that already one copy of *BAC*^*shrub*^ led to a significant rescue, allowing the development of *lgd* mutant flies beyond their normal time of death at early pupal stage to the pharate adult stage (Fig. 7A). The analysis of the discs revealed that the presence of an additional copy of *shrub* suppresses the ectopic activation of the Notch pathway nearly completely (Fig. 7B, B’). The presence of two extra copies of *BAC*^*shrub*^ allowed the development of the *lgd* null mutants even further to normal looking, albeit sterile adult flies (Fig. 7A). Wing imaginal discs of these rescued *lgd* mutants were normal in size and expression of Wg or *Gbe+Su(H)* (Fig. 7C, C’).

The TEM analysis revealed that the enlargement of the MEs characteristic for *lgd* mutants was partially rescued: The average diameter of the MEs was reduced to a value between that of wt and *lgd* mutants (Fig. 2C, D). In contrast, the presence of two copies of *BAC*^*shrub*^*-mut2* did not rescue the *lgd* mutant phenotype. Instead, the flies died even earlier, in fact already before the third instar larval stage (Fig. 7A). Importantly, an increase in the levels of Vps20 (*BAC*^*vps20*^), the initiator of Shrub polymerisation at the LM, did not rescue the *lgd* mutant phenotype, even if *BAC*^*vps20*^ was present in two copies. Instead, it resulted in an earlier time of death of *lgd* mutants during the early third larval instar stage, if two copies were present (Fig. 7A, D–E’).

Furthermore, by adding two copies of *BAC*^*shrub*^*-CHMP4B*, we found that also the human ortholog CHMP4B can rescue *lgd* mutants, albeit to a lesser extent than BAC^*shrub*^. The rescued flies nearly developed to the pharate adult stage (Additional file 5: Figure S5A). In the wing discs of these partially rescued flies, a weak ectopic activation of Notch was observed (Additional file 5: Figure S5E, E’, arrow in E, compare with B–C’). In contrast, one copy had only little effect on the time of death (Additional file 5: Figure S5A) and only a weak effect on the ectopic activation of the Notch pathway (Additional file 5: Figure S5D, D’, compare with B-C’). A clear effect was only observed on the expression of Wg, which requires high Notch activity and is therefore particularly sensitive to changes in Notch activity (Additional file 5: Figure S5D, D’).

Collectively, the results further confirm the notion that insufficient activity of *shrub* is present in *lgd* mutants. The reduction in activity can be compensated by elevation of Shrub levels, achieved by the addition of extra copies of *shrub*, or its human ortholog *CHMP4B* to the genome.

### Lgd regulates the activity of Shrub

The observed insufficient activity of *shrub* in *lgd* mutants is caused either by a reduction in expression of *shrub* or, post-transcriptionally, by a reduction of the activity of the protein. To discriminate between these possibilities, we monitored the effect of loss of *lgd* function on the levels of Shrub in *lgd* mutant cell clones in imaginal discs. Using clonal analysis, no difference in the levels of Shrub between the *lgd* mutant and wild-type cells was observed (Fig. 5I–K’). Hence, Lgd is required for the full activity of Shrub, but not for its expression.

### A small fraction of Lgd is associated with the LM of the ME

**Fig. 8:**
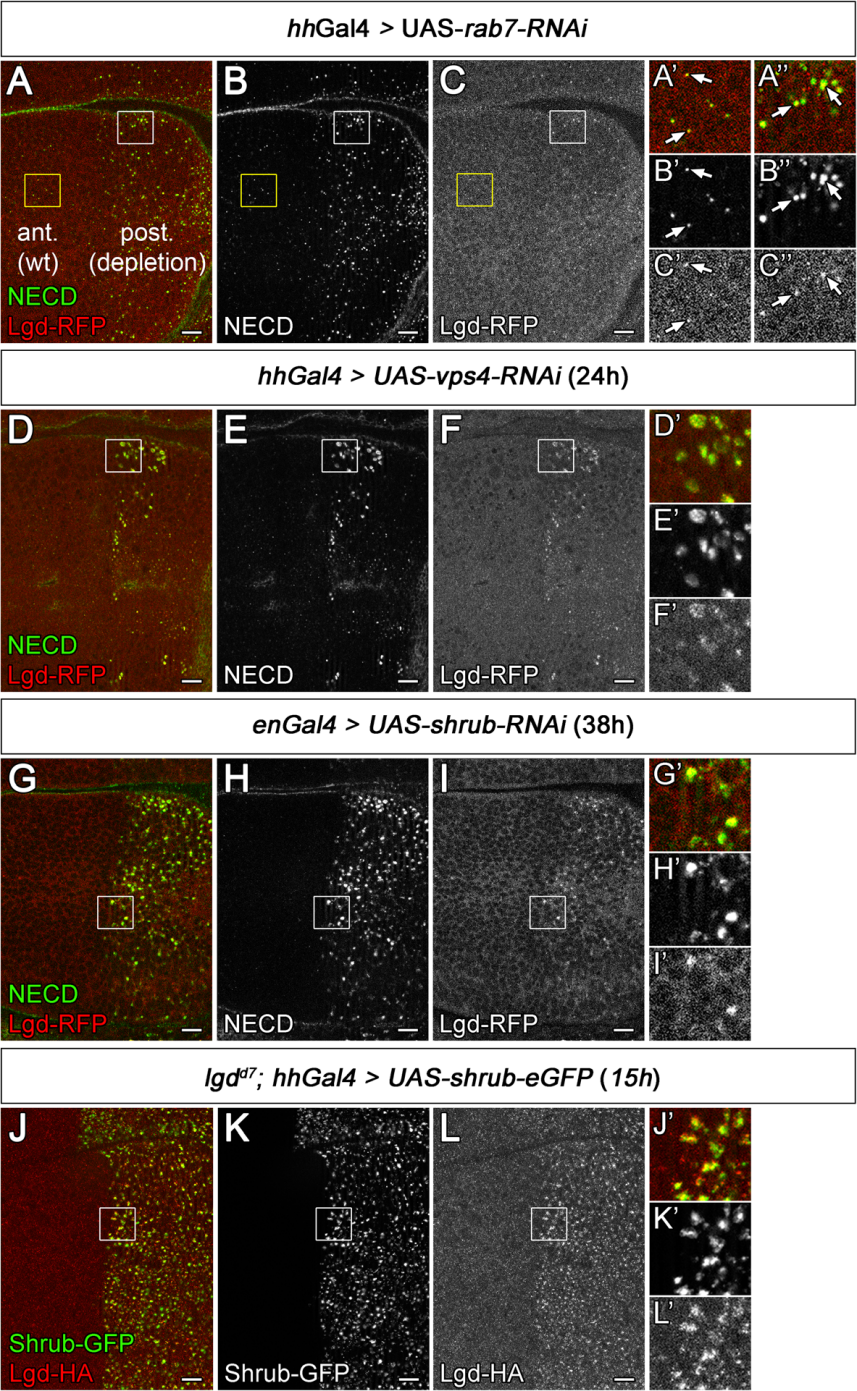
Lgd cycles between the cytosol and the LM of the ME. (**A’**–**C”**) Depletion of Rab7 in the posterior compartment of a wing disc using an UAS *rab7-RNAi* construct continuously driven by *hh*Gal4. (**A’**–**C’**) Magnification of yellow outlined area, located in the wild-type anterior compartment and (**A”**–**C”**) of the white box, located in the posterior Rab7-depleted compartment of the same wing discs shown in (**A**–**C**). The arrows point to Notch-positive MEs that are associated with Lgd-RFP. (**D**–**F’**) RNAi mediated depletion of *vps4* function in the posterior compartment of the wing imaginal disc. *hh*Gal4 *tub*Gal80^ts^ was used to drive expression of UAS *vps4-RNAi* for 24 h. The depletion results in an enlargement of Notch-positive MEs. Magnification of the region boxed in (**D**–**F**) is shown in (**D’**–**F’**). (**G**–**I’**) Depletion of *shrub*-RNAi for 38 h using *en*Gal4 *tub*Gal80ts leads to localisation of Lgd at enlarged ME. Magnification of the area boxed in (**G**–**I**) is shown in (**G’**–**I’**). (**J**–**L’**) Expression of the dominant-negative Shrub-GFP with *hh*Gal4 for 15 h. Lgd-HA accumulates on the enlarged MEs together with Shrub-GFP. Magnification of the region boxed in (**J**–**L**) is shown in (**J’**–**L’**). Scale bars (**A**–**L**) 10 μm. At least ten wing imaginal discs were analysed for each genotype

Previous work reports that Lgd is distributed in diffuse punctae in the cytosol of imaginal disc cells with no obvious association with endosomes [43]. In agreement with this finding, no obvious association with endosomes was also observed for HA- and GFP-tagged variants of Lgd expressed with the Gal4 system [8]. Altogether, these results indicated that Lgd is not obviously associated with endosomes. These in vivo results contrasted in vitro studies that showed that the C2 domain of Lgd can bind to phospholipids characteristic for the endosomal and cell membrane, although different studies identified different phospholipids as binding partner [13, 16]. In addition, the mammalian orthologs of Lgd could be detected in the membrane fraction in fractionation experiments [15]. To further investigate the issue of association of Lgd with endosomes, we used a C-terminal HA- or RFP-tagged Lgd expressed under the endogenous promoter (*lgdP*-*lgd-RFP*, *-HA*, [8]). Also, these variants were diffusely expressed in small dots within the cytosol. Careful scrutinization revealed that a few of these punctae were Notch-positive endosomes (Fig. 8A–C, A’–C’, anterior unmanipulated half, square area outlined in yellow, arrows). To reveal a possible association of Lgd with MEs with more clarity, we depleted the cells of the posterior half of the wing imaginal disc of the activity of Rab7 by expression of an UAS *rab7-RNAi* construct with *hh*Gal4 (Fig. 8A–C, A”–C”, posterior). As shown here, Rab7 depletion causes a dramatic enlargement of the Notch-positive endosomes, but does not affect ILV formation [32] (Fig. 3E’, G). The depletion had little effect on the general distribution of Lgd-RFP (Fig. 8A, C and C”). However, the analysis of the enlarged endosomes revealed an association of Lgd punctae with endosomes more clearly (Fig. 8A–C”, arrows). Hence, it appears that a small fraction of Lgd is associated with the ME.

### Loss of *vps4* function results in an accumulation of Lgd on the LM of the ME

One explanation for the generally poor association of Lgd with endosomes in wild-type cells is that Lgd cycles between the cytosol and the endosomal membrane with only a short resident time on the membrane. This has been observed for Snf7 in yeast and also for Shrub [30, 44]. We tested this possibility by monitoring the distribution of Lgd-RFP in cells where the ESCRT-mediated process was stalled at different stages. We used Notch staining to reveal MEs. As reported previously, depletion of Vps4 resulted in a strong accumulation of Shrub at Notch-positive enlarged MEs [30]. We here observed also a strong accumulation of Lgd (Fig. 8D–F’). Albeit weaker, the accumulation of Lgd at the ME was also observed in cells depleted of *shrub* or *vps20* (Fig. 8G–I’, Additional file 6: Figure S6A-C’). The different depletion experiments are not easily comparable, since the efficiency of the RNAi lines and the strength of depletion is not known. We therefore believe that Shrub is involved in the recruitment of Lgd to the LM, but cannot rule out that Shrub-independent recruitment mechanisms might also exist. It is to mention here that Lgd contains a C2 domain which can bind to phospholipids [13, 16]. Therefore, Lgd might be able to locate to membranes without the interaction with Shrub.

To further confirm the involvement of Shrub in the recruitment of Lgd to the LM of the ME, we expressed Shrub-GFP, which is a dominant-negative version of Shrub [42]. It accumulates at the endosomal membrane of MEs (Fig. 8J, K, J’, K’). These enlarged MEs were also strongly positive for Lgd (Fig. 8L–L’). This result supports the notion that Shrub can recruit Lgd to the LM of the ME. Altogether, the results suggest that Lgd cycles together with Shrub between the cytosol and the LM of the ME.

To test whether also mammalian LGD1 behaves similar to Lgd, we monitored the cellular distribution of LGD1-HA expressed under the promoter of *lgd* (*lgdP*-*LGD1*) in cells that expressed Shrub-GFP [15]. We found a similar accumulation of LGD1 at the LM of the ME (Additional file 6: Figure S6D-J). Hence, at least in imaginal disc cells of *Drosophila*, LGD1 also cycles between the cytosol and the LM.

### LGD1 binding does not influence the conformation of CHMP4B

CHMP4B/Shrub is supposed to exist in an open conformation that polymerises at the LM of the ME, and in a monomeric closed conformation in the cytosol, which is unable to polymerise. We have previously found that the binding of Shrub to Lgd and its homo-polymerisation is mutually exclusive [6]. It is possible that the binding of Lgd to Shrub prevents its polymerisation by promoting the closed conformation, in other words that Lgd acts as a chaperone of Shrub. To test this possibility, we adapted an established cysteine-based cross-linking strategy to directly monitor the conformational state of Snf7 for its mammalian ortholog CHMP4B [45]. We then used this assay to analyse the consequences of LGD1 binding for the conformational state of CHMP4B. Both of these human orthologs can rescue the mutant phenotype of the respective mutants in *Drosophila* ([15] and this work). Taking the advantage that CHMP4B lacks cysteines, we introduced them at two positions that have been previously proven suitable to detect conformational changes via the formation of disulfide bonds induced by oxidation with Cu^2+^Phenanthroline [45] (CHMP4B^A54C; L180C^=CHMP4B^redox^). The functionality of *CHMP4B*^*redox*^ was shown in rescue experiments in *Drosophila* where it can rescue the phenotype of *shrub* mutant cell clones (Additional file 7: Figure S7A-A”) and in Co-IP experiments that show that LGD1 binds CHMP4B^redox^ (Additional file 7: Figure S7B). For the rescue experiments, CHMP4B^redox^ was expressed at an endogenous level under control of *shrub* promoter (*shrubP-CHMP4B*^*redox*^) [4].

In our experiment, recombinant CHMP4B^redox^ was oxidised under native conditions to freeze the amount of CHMP4B existing in the closed form in solution and then subjected to non-reducing denaturing SDS-PAGE to separate the faster migrating fixed closed form from the slower migrating open form (Additional file 8: Figure S8A). We found that, in the buffer solution, the closed form prevails, even in the absence of oxidation, suggesting that the majority of CHMP4B^redox^ adopts the closed conformation (Additional file 8: Figure S8B, lane 1: ca 74%). Nevertheless, a significant fraction is in the open conformation (Additional file 8: Figure S8B, lane 1: ca 26%). The addition of the reducing agent DTT severs the cysteine-mediated disulfide bonds and results in the loss of the faster migrating closed form and a corresponding increase in the concentration of the open form (Additional file 8: Figure S8B, lane 2: 98%). We exploited this behaviour to test the stability of the two conformations of CHMP4B^redox^. We reduced the probe with DTT to get rid of the closed form and incubated subsequently 30 min at room temperature before fixing the fraction that has adopted the closed form in this time interval. We found that 29.9% have adopted that closed form, indicating that CHMP4B^redox^ changes between the two conformations (Additional file 8: Figure S8B, lane 4: 29.9% closed and 70.1% open conformation).

The addition of LGD1 to CHMP4B^redox^ did not change the relative abundance of the two conformations of CHMP4B^redox^, indicating that the presence of LGD1 does not influence the conformation of CHMP4B^redox^ in solution (Additional file 8: Figure S8B, lanes 5–7, compare with lanes 1, 3 and 4). Hence, it appears that Lgd does not act as a chaperone.

## Discussions

Lgd is an evolutionary conserved protein that physically interacts with the ESCRT-III core component Shrub/CHMP4 [5, 8]. The loss of function of each gene results in the uncontrolled activation of the Notch pathway in *Drosophila*. It was not clear how this activation occurs. It has been previously shown that the activation of the Notch pathway in *lgd* mutants requires the fusion of the ME with the lysosome and also depends on the activity of the vATPase, which is required for the acidification of MEs and the activation of the acidic hydrolases in its lumen [7]. These findings combined with the intimate relationship between Lgd and Shrub/CHMP4 led to the working hypothesis that, in *lgd* mutants, incorporation of Notch into ILVs must be disturbed, resulting in the remaining of a fraction of it on the LM of the ME. After fusion with the lysosome, the activated luminal hydrolases degrade the ECD of this fraction of Notch, thereby creating a NEXT-like intermediate that can be recognised and cleaved by γ-secretase to release NICD into the cytosol [7]. The escape of Notch from ILV formation is a prerequisite for the activation, as it is an absolute prerequisite for the released NICD to translocate into the nucleus. Recently, the analysis of *lgd* mutants with a Notch variant that is doubly tagged with GFP and Cherry in its ICD provided evidence that a fraction of Notch remains in the LM of *lgd* mutant MEs [23]. Here, we provide further experimental evidence that this LM fraction exists and that it is this fraction that is activated. We found that the incorporation of Notch into ILVs, forced by over-expression of Su(dx), suppresses the ectopic endosomal activation of the Notch pathway in *lgd* mutants without affecting the normal ligand-induced activation. Likewise, the loss of Dx, the antagonist of Su(dx), suppresses the ectopic activation. Thus, removing Notch from the LM of *lgd* mutant MEs abolishes the ectopic activation. The findings also underscore that it is important for a cell to assure that Notch is correctly incorporated into ILVs before fusion with the lysosome to avoid uncontrolled activation of the pathway. It has to be mentioned that the defect caused by *lgd* loss of function is probably of general nature, since the degradation of several TMPs is delayed [7].

It was unclear how the fraction of Notch escapes the incorporation into ILVs in *lgd* mutants. We here show that a reduction in the activity of Shrub is causative. (1) The temporal depletion of *shrub*, as well as analysis of a hypomorphic *shrub* situation achieved by the partial rescue of *shrub* mutants with one copy of *BAC*^*shrub*^*-CHMP4B,* caused phenotypes that resemble that of *lgd* mutants. (2) Introduction of additional copies of *shrub* in the genome rescued the *lgd* mutant phenotype. These results clearly indicate that loss of *lgd* function constitutes a state of reduced Shrub activity. The reduction is probably below 50%, because *shrub* heterozygous flies are normal and fertile, while *lgd* mutants die in the early pupal stage.

It is not clear how a fraction of Notch escapes its incorporation into ILVs in *lgd* mutants because of a reduction in the activity of ESCRT-III/Shrub. However, it has been recently shown that proper ESCRT-III assembly is required for the clustering of cargo: if the activity of ESCRT-III is disturbed, e.g. by loss of the activity of the auxiliary ESCRT-III factor Ist-1, cargo fails to cluster [46]. Moreover, the depletion of the Shrub ortholog of *C. elegans*, Vps32, results in an increase of the ESCRT-0 domain, which recruits Clathrin to the sites of invagination and therefore leads to electron-dense microdomains seen in the TEM [35, 46]. Conversely, the loss of ESCRT-0 function results in the loss of the ESCRT-containing electron-dense microdomains [35]. We here found that the size of the ESCRT-containing electron-dense microdomains of *lgd* mutant MEs is increased. Moreover, our results also indicate that the rate of ILV formation is concomitantly slightly decreased in comparison to wild-type cells. These observations suggest that in *lgd* mutants, the activity of Shrub is reduced, which results in an increase in the length of the ESCRT-containing electron-dense microdomains and a decrease in the rate of ILV formation. Somehow, these changes in ESCRT-III activity cause the escape of cargo, among it Notch. Recent work indicates that ILV formation is more dynamic than anticipated, requiring the constant assembly and disassembly of several Shrub/Snf7 filaments at a time [34]. This dynamic behaviour induces cargo crowding and the invagination of the LM of the ME. Hence, a slight reduction in the activity of Shrub can have a large impact on the dynamic equilibrium that is required for the proper incorporation of cargo.

Our findings are in agreement with the recently discovered role of LGD1/CC2D1B in coordinating ESCRT-III polymerisation during nuclear envelope reformation [16]. Also, in this process, the reformation of the envelope by ESCRT-III eventually occurs in the absence of LGD1, but is delayed. The delay causes morphological defects of the nuclear lamina.

How can a decrease in Shrub activity be explained in *lgd* mutants? We have previously shown that Shrub homo-polymerisation and Shrub binding to Lgd is mutually exclusive and that the addition of Lgd to the truncated activated form of Shrub prevents its spontaneous polymerisation in in vitro experiments [4, 6]. A similar behaviour has been found for the corresponding mammalian orthologs LGD2 and CHMP4B, which bind each other in a 1:1 ratio in in vitro experiments [5]. Full-length Vps32 of *C. elegans*, which is the ortholog of Shrub, can polymerise spontaneously in solution [47]. We here found that a considerable fraction of full-length CHMP4B (ca 26%, Additional file 8: S8B, lane 1) is in its open form in solution, which is the precondition for polymerisation. Moreover, we find a dynamic equilibrium between the two forms. Assuming this holds true also for monomeric Shrub in the cytosol, the fraction of Shrub in its open conformation is endangered to inappropriately polymerise, unless the negative surface is not obscured by binding to Lgd. Having found that LGD1 does not act as a chaperone of CHMP4B, which assures that all of cytosolic CHMP4B is in its closed form, it is plausible that it just binds to the monomeric form of Shrub in the cytosol via its DM14 domains and, by obscuring the negative surface required for polymerisation, prevents inappropriate polymerisation of Shrub in the cytosol. In the absence of *lgd* function, the Shrub fraction in the open form would inappropriately polymerise in the cytosol and therefore not available for the appropriate polymerisation at the LM of the ME. Hence, the net activity of Shrub would be decreased in *lgd* mutants, although its concentration has not changed. This is what we observed here. This scenario also explains why addition of extra copies of Shrub rescues the *lgd* mutant phenotype, since it compensates for the loss of available Shrub monomers trapped in cytosolic non-functional polymers. However, we did not observe obvious Shrub aggregates in *lgd* mutant cells using antibody staining. This can be explained by either a rapid degradation of the aggregates, the amount or size of aggregates which are not detectable by the staining.

Here, we found that Lgd accumulates together with Shrub on the LM of MEs of *vps4*-depleted cells, although it is hardly detected on the LM of wild-type MEs. Thus, Lgd might assist Vps4 in the disassembly of the Shrub polymer at the LM by receiving the removed Shrub monomers already at the LM and subsequently stay in complex with the monomers to prevent their inappropriate polymerisation in the cytosol.

Our results support the notion that the interaction between Shrub and Lgd is conserved in mammals in vivo. We found that the addition of two copies of *BAC*^*shrub*^-*CHMP4B* to *shrub* mutant flies, which normally die during embryogenesis, allowed the development of normal looking adult flies. One copy allowed the development up to the pharate adult stage. The imaginal discs of the partially rescued flies with only one copy displayed a weak *lgd* mutant-like phenotype with ectopic activation of the Notch pathway, which can be strongly enhanced by the removal of one copy of *lgd*. These findings indicate that CHMP4B is a functional ortholog of Shrub that also requires the interaction with Lgd to function properly in vivo.

## Conclusions

In conclusion, our data show that Lgd/CC2D1 is required for the full activity of Shrub/CHMP4. Its loss of function results in the insufficient incorporation of the Notch receptor into ILV due to the reduction in the activity of ESCRT-III. The fraction that remains on LM of the ME is activated after fusion with the lysosome. Lgd is not affecting the conformation of Shrub/CHMP4 and is therefore not acting as a chaperone.

## Methods

### Fly stocks

The following fly stocks were used during this analysis. Mutants: *lgd*^*SH495*^ and *lgd*^*d7*^ [13, 14, 48], *Su(dx)*^*sp*^ [28], *dx*^*152*^ [27], *shrub*^*4-1*^ *FRTG13* [42]. UAS-constructs: UAS *Myc-shrubΔauto and* UAS *Myc-shrub* [8], UAS *shrub-GFP* and UAS *shrub-*RNAi [42], UAS *vps4-*RNAi (VDRC #35126), UAS *vps20-RNAi* (VDRC #26387), *Gbe+Su(H)-lacZ* [49], *NRE-pGR* [50], *lgdP*-*lgd-HA* and *lgdP*-*lgd-RFP* [51], *lgdP*-*LGD1* [15], *hh*GAL4 [52], *tub-rab7-YFP* [53]. This work: *shrubP-CHMP4B*^*redox*^, *BAC*^*shrub*^, *BAC*^*shrub*^*-CHMP4B, BAC*-^*shrub*^*-cDNA*, BAC^*vps20*^.

### Constructs

#### For protein purification

Full-length human *CHMP4B*^*A54C;L180C*^ (*CHMP4B*^*redox*^), synthesised by GenScript (USA), was cloned into the pQE-30 Xa plasmid. *CHMP4B*^*redox*^ and *LGD1* were amplified by PCR using constructs in pQE-30 Xa as a template. Ligation of KpnI and HindIII digested *CHMP4B*^*redox*^ and KpnI and EcoRI digested *LGD1* with pBAD-HisB vector resulted in the generation of pBAD-His6-EK-*CHMP4B*^*redox*^ and pBAD-His6-EK-*LGD1*. The coding sequence for N-terminal His6 and EK was replaced by a His10 and TEV-cleavage site using PCR to create the AgeI-pBAD-His10-TEV-KpnI product.

Human *CHMP4B*^*redox*^ was cloned into the pattB-*shrubP* vector by PCR using primer containing NotI and KpnI cleavage sites and pQE-30 Xa-*CHMP4B*^*redox*^ as a template.

#### For expression in *Drosophila*

The *shrubP*-*CHMP4B*^*redox*^ construct is based on rescue constructs described previously [4]. This construct was generated containing the CHMP4B cDNA flanked by the proximal *shrub* genomic elements (510 bp upstream and downstream of start and stop codon of the *shrub*, template *BAC*^*shrub*^ CH422-47O20). *BAC*^*shrub*^ (CH422-47O20) and *BAC*^*vps20*^ (CH322-09O08) were ordered at BACPAC Resources Center. The constructs are inserted into the genomic attP-landing site at 86Fb by Bestgene [54].

*BAC*^*shrub*^*-CHMP4B* and *BAC*^*shrub*^*-cDNA* are based on *BAC*^*shrub*^. The transcription unit of BAC^*shrub*^ is replaced by a cDNA of *shrub* (*BAC*^*shrub*^*-cDNA*) or by a cDNA of CHMP4B (*BAC*^*shrub*^*-CHMP4B*) performing recombination-mediated genetic engineering [55].

### Immunohistochemistry and light microscopy

For the antibody staining of wing imaginal discs, standard protocols were used [56]. The following antibodies were obtained from the Developmental studies Hybridoma Bank: mouse anti-Wg 4D4 (1:500), mouse anti-NECD (Notch extracellular domain) C458.2H (1:100), mouse anti-Discs large 4F3 (1:500) and rat anti-DE-cadherin DCAD2 (1:50), rabbit anti-Rab7 (1:50) [57], rabbit anti-ßGal (1:1500) (Cappel), rat anti-HA 3F10 (1:500) (Roche), rabbit anti-Shrub (1:125) [30] and rat anti-Crumbs (Crb) (1:500) [58]. Fluorochrome-conjugated antibodies were purchased from Invitrogen/Molecular Probes.

Larvae were dissected under a Zeiss Stemi 2000-C stereomicroscope. Images of wing imaginal discs were obtained with a Zeiss AxioImager Z1 using the Zeiss Apotome device.

### Transmission electron microscopy

Wing discs were fixed in 2.5% glutaraldehyde, washed in 100 mM phosphate buffer and post-fixed in 2% osmium tetroxide in phosphate buffer for 1 h on ice. The specimens were dehydrated in ethanol and embedded in araldite using acetone as an intermediate solvent. Thin sections were stained with 2% uranyl acetate and lead citrate. Sections were observed under an EM 902 (Zeiss) microscope at 80 KV.

For the statistical analysis of MVBs, their area, electron-dense material/area and the size of the ESCRT-containing electron-dense microdomains were measured using processing the image processing software “Fiji”. The obtained data were statistically analysed using Microsoft Excel and GraphPad Prism 7.0d.

### Quantitative analysis of MVBs

To quantify the ILV content, electron-dense material within the lumen of photographed MVBs was measured by utilising a self-written macro for the image processing software “Fiji” [59]. This analysis included the following steps. MVBs were manually outlined as regions of interest (ROIs) and stored for analysis. Each individual MVB was cropped first. To reduce noise and preserve boundaries as much as possible, a median filter of 2 pixel range was applied. MVBs were delimitated using the “Huang” algorithm [60] and converted into a binary mask, highlighting areas of electron-dense material of ILVs. The areas of all masked ILVs were summed up and divided by the area of the whole corresponding MVB, resulting in the quotient of ILV material per MVB in percent. Colourized overlays of estimated ILV areas with the original electron microscopy image were revisited for quality control after the analysis was finished. Area quotients of several MVBs are collected and plotted in a box-whisker diagram. The obtained data were statistically analysed using GraphPad Prism 7.0d.

### Protein expression

Coding sequences of human *CHMP4B*^*A54C;L180C*^ (*CHMP4B*^*redox*^) and *LGD1* were transformed into LOBSTR strain [61] to inoculate the preculture consisting of 300 ml LB Amp media and one single bacterial colony. The main culture (2 l pro construct) was grown initially at 37 °C until a cell density of OD_600_ 2.0–3.0 was achieved. Protein expression was induced by the addition of L-(+)-Arabinose (Sigma-Aldrich; 10 mM). After induction, CHMP4B was expressed for 4 h at 37 °C. Expression of LGD1 occurred at 18 °C for 3 h. Bacterial pellets were centrifuged at 5000×*g*, shock frozen in liquid nitrogen and stored at − 70 °C until the purification.

### Protein purification

All proteins were purified using HisTalon Gravity Columns (Takara Bio Inc). The purification of His-LGD1 occurred under native conditions, whereby bacterial pellets were resuspended in lysis buffer (50 mM NaH_2_PO_4_, 300 mM NaCl, 5% glycerol, lysozyme (1.5 mg/ml), PMSF (1 mM), PIC (Sigma-Aldrich P8340; 1:200), DNaseI (Thermo Scientific; 10 U/ml) and RNaseA (Thermo Scientific; 0.01 mg/ml); pH 7.4) and incubated for 30 min at 4 °C under permanent rotation. After the sonification (4 × 30 s per pulse with 2-min pauses on ice), the lysate was centrifuged for 1 h at 48,400×*g* at 4 °C. Additionally, the column was loaded with a clarified sample and incubated 1 h under permanent rotation at 4 °C. Afterwards, the column was washed twice with Wash Buffer I (50 mM NaH_2_PO_4_, 300 mM NaCl; 5% glycerol, pH 7.4) and with Wash Buffer II (50 mM NaH_2_PO_4_, 300 mM NaCl, 5% glycerol, 20 mM imidazole; pH 7.4). His-LGD1 was eluted from the column by incubating with 2.5 ml of the elution buffer (50 mM NaH_2_PO_4_, 300 mM NaCl, 150 mM imidazole; pH 7.4) for 30 min under permanent rotation.

The purification of His-CHMP4B^redox^ required denaturing conditions due to the inaccessibility of the added His-Tag in the native protein. Therefore, all purification steps occurred under the addition of 8 M urea to each buffer. Because of the presence of 8 M urea, the lysis buffer for CHMP4B did not require the addition of lysozyme, PMSF, PIC, DNaseI and RNaseA.

### Buffer exchange and concentration of proteins

The refolding of CHMP4B^redox^ was performed by buffer exchange using PD10 columns (GE Healthcare). The column was washed with 25 ml dH_2_O and equilibrated with Buffer A (20 mM HEPES, pH 7.4 and 150 mM NaCl). Additionally, 2.5 ml protein sample was loaded onto the column. Protein was eluted by adding 3.5 ml Buffer A.

The concentration of proteins was performed using Amicon Ultra-15 centrifugal filters (Merck Millipore; 10 MWCO for CHMP4B^redox^) and Vivaspin 2 (Sartorius; 30 MWCO for LGD1) according to the manufacturer’s information. Proteins were stored at 4 °C.

### Dicysteine oxidative cross-linking assay

The analysis of conformational changes of CHMP4B was performed by modifying an established cysteine-based cross-linking strategy [32]. Samples were treated with 10 mM DTT for 20 min at 37 °C to generate a CHMP4B population consisting of only open conformation. Afterwards, LGD1 was added to the sample and the proteins were incubated at RT for 30 min. The fixation of the current conformation was achieved upon the oxidation with Cu^2+^Phenanthroline solution (200 mM 1,10-Phenanthroline in ethanol and 50 mM CuSO4 in Ionic Buffer consisting of 150 mM potassium acetate, 5 mM magnesium acetate, 250 mM sorbitol, 20 mM HEPES pH 7.0; 1:1 ratio).

After 15-min incubation on ice, the reaction was stopped by the addition of 15 mM EDTA. The samples were mixed with 4x non-reducing Laemmli buffer before being analysed on SDS-PAGE. The gel was stained in colloidal Coomassie-Brilliant-Blue solution and analysed using GelAnalyzer (GelAnalyzer 19.1 (www.gelanalyzer.com) by Istvan Lazar Jr., PhD, and Istvan Lazar Sr., PhD, CSc).

### Co-immunoprecipitation

Purified CHMP4B^redox^ and LGD1 were incubated with rabbit αCHMP4B antibody (C12: sc-82556; Santa Cruz Biotechnology; 1:250) overnight at 4 °C under permanent rotation following by the incubation with Protein G Sepharose beads (GE Healthcare; 40 μl beads pro sample) under the same conditions. Afterwards, the sample was washed with Wash Buffer I (see the “Protein purification” section) and incubated with 30 μl 4x Laemmli buffer for 10 min at 95 °C. Proteins were detected via Western blot using mouse αHis (pentaHis:34660; Qiagen; 1:3000) antibody and rabbit αCHMP4B antibody (C12: sc-82556; Santa Cruz Biotechnology; 1:2000).

## Supplementary Information

**Additional file 1: Figure S1.** Clonal analysis of *Su(dx)*. Clones are labelled by the absence of GFP. (A) Expression of the Notch activity reporter *Gbe+Su(H)* in a control wild-type disc. (B-C”’) A disc bearing *Su(dx)*^*sp*^ clones, which is kept on 29 °C to maximise the mutant phenotype. Notch activity is revealed by the expression of *Gbe+Su(H)*. (B-B”) Overview of the disc. (C-C”’) Magnification of the wing area. A large *Su(dx)*^*sp*^ mutant clone is outlined in white. No ectopic expression of *Gbe+Su(H)* is observed. (D-D”’) The wing area of a disc bearing *lgd* mutant clones, labelled by the absence of GFP. One large *lgd* mutant clone is outlined in white. The expression of Wg and *Gbe+Su(H)* is clearly activated ectopically (in all clones). Scale bars: (A-B”) 200 μm; (C-D”’) 50 μm. At least ten wing imaginal discs were analysed for each genotype.

**Additional file 2: Figure S2.** Genetic interactions between *lgd* and *Su(dx)*. (A) Time of death of the single and double mutants during development. (B-C’) The phenotype of wing discs of *lgd Su(dx)* double mutants at 18 and 25 °C, respectively. The arrow in (B, B’) points to the suppression of Notch activation in the dorsal compartment of the wing anlage. The arrow in (C) highlights the formation of an additional winglet often seen upon strong Notch activation. For further information see text. Scale bars: (B-C’) 200 μm. At least ten wing imaginal discs were analysed for each genotype.

**Additional file 3: Figure S3.** Analysis of MEs/MVBs of cells expressing Shrub or ShrubΔauto. Comparative analysis of the consequences of expression of Myc-Shrub and Myc- ShrubΔauto. Restricted expression of these variants in the posterior (post.) compartment for 13 h is achieved by using *hh*Gal4 combined with *tub*Gal80^ts^. (A-B’) In contrast to expression of Myc-Shrub (A-A’), the expression of Myc-ShrubΔauto (B-B’) causes the formation of enlarged Rab7-YFP (*tub-Rab7-YFP*) and Notch positive MEs. Magnification of the boxed area in (A-B’) explicitly shows that Notch positive structures are MEs (ant. arrows and post. arrowheads). (C-F) TEM analysis of the discs shown in (A, B). (C, D) Transverse semi-sections of analysed wing imaginal discs. (C’-D”) Representative TEM images of the MEs/MVBs of the wild-type anterior (ant.) control cells and posterior cells expressing the Shrub variants. (E) Measurement of the area of the MEs/MVBs. The average area of MEs of Myc-ShrubΔauto expressing cells is significantly increased in comparison to the anterior control cells (blue). However, there is no significant change in the MVB area upon over-expression of full-length Shrub for 13 h (light-green). (F) Pixelwise Quantification of the electron dense material of the MEs/MVBs. Quotient of ILV/ electron dense material per MVB area [%] are collected and plotted in a box plot. The ILV content within MVBs of cells expressing Myc -Shrub is similar to wild-type, whereas it is significantly reduced in MVBs of cells that express Myc-ShrubΔauto. [(E, F) Over-expression of Myc-Shrub (highlighted in light-green): wt control (ant.): *n* = 52 MVBs, Myc-Shrub (post.): *n* = 46 MVBs; Over-expression of Myc-ShrubΔauto (highlighted in blue): wt control (ant.): *n* = 25 MVBs, Myc-ShrubΔauto (post.) *n* = 33 MVBs; (E) Over-expression of Myc-Shrub (light-green): Mann Whitney test, Two-tailed; Over-expression of Myc-ShrubΔauto (blue): unpaired t-test; (F) Over-expression of Myc-Shrub and of Myc-ShrubΔauto: both unpaired t-test; box plots: whiskers: 5–95 percentile; mean shown as”+”; *p* < 0.001 (***)]. Scale bars: (A-B’) 10 μm; (C’- D”) 250 nm. (A-B’) At least ten wing imaginal discs were analysed for each genotype.

**Additional file 4: Figure S4.** Rescue of *shrub* null mutants with *BAC*^*shrub*^*-CHMP4B*. (A, B) Time of death during development of various genotypes rescued by various copies of *BAC*^*shrub*^*, BAC*^*shrub*^*-cDNA or BAC*^*shrub*^*-mut2* (A), or *BAC*^*shrub*^*-CHMP4B* (B). (C-F’) Wing imaginal discs of flies rescued with various copies of *BAC*^*shrub*^*-CHMP4B*. (C, C’) The Notch activity detected by Wg or *Gbe+Su(H)* in presence of two copies *BAC*^*shrub*^*-CHMP4B* is comparable to wild-type, even in *lgd* heterozygosity (D, D’). (E, E’) A partial rescue is observed if only one copy of *BAC*^*shrub*^*-CHMP4B* is present. A few cells near the D/V-boundary show an ectopic activation of Wg (E, arrow). (E’) *Gbe+Su(H)* is slightly ectopically expressed. (F, F`) This ectopic activation is enhanced, if one copy of *lgd* is removed in the partial rescued discs. The observed phenotype resembles that of strong *lgd* mutants (compare with Fig. 1C, C’). (G-J) Z-stack through the wing pouch of wing imaginal discs: Comparative Analysis of the apicobasal polarity of wild-type wings discs and *shrub* mutant discs partially rescued with one copy of *BAC*^*shrub*^*-CHMP4B*. (J) Induction of *shrub* mutants clones in presence of one copy of *BAC*^*shrub*^*-CHMP4B.* Mutants clones are labelled by the absence of GFP. (G-J) The apicobasal polarity is unaffected in comparison to the wild-type, indicated by the normal localisation of E-cad, Dlg and Crb. Scale bars: (C-F’) 200 μm; (G, H) 50 μm; (G-J) 10 μm. (C-J) At least ten wing imaginal discs were analysed for each genotype.

**Additional file 5: Figure S5.** Addition of copies *BAC-shrub*^*CHMP4B*^ partially rescues *lgd* mutant flies. (A) Summary of the time of death of *lgd* mutant flies rescued with one or two copies of *BAC*^*shrub*^*-CHMP4B.* (B-E`) The expression of Notch targets in the corresponding wing imaginal discs reveals that the degree of rescue of *lgd* mutants is proportional to the number of copies of *BAC*^*shrub*^*-CHMP4B* present in the genome. Scale bars: (B-E’) 200 μm. At least ten wing imaginal discs were analysed for each genotype.

**Additional file 6: Figure S6.** Lgd cycles between the cytosol and the LM of the ME. (A-C’) Depletion of the function of *vps20* in the posterior compartment of the wing discs results in the formation of strongly enlarged Notch positive MEs, but in only weak accumulation of Lgd-RFP on the LM. Magnification of the area boxed in (A-C) is shown in (A’-C’). (D-J) Expression of Shrub-GFP in the posterior compartment of a *lgd* mutant wing disc rescued with LGD1 expressed under control of the *lgd* promoter results in the accumulation of LGD1-HA on enlarged Notch positive MEs. This shows that also LGD1 can be recruited to the ME by Shrub. (E-J) Magnification of the region boxed in (D) is shown in (E-J). Scale bars: (A-D) 10 μm. At least ten wing imaginal discs were analysed for each genotype.

**Additional file 7: Figure S7.** Testing the functionality of *shrubP-CHMP4B*^*redox*^. (A-A”) Induction of *shrub* mutant clones in the presence of one copy of *shrubP-CHMP4B*^*redox*^ in the genome. Clones are labelled by the absence of RFP and outlined with the white line in (A’, A”). The arrow in (A) points to a large clone shown at higher magnification in (A’, A”). Loss of *shrub* function in wing disc cells is normally cell lethal (see Fig. 5A–E). The presence of *CHMP4B*^*redox*^ prevents death of the *shrub* mutant cells and leads to a normal appearance of the MEs in the mutant cells. This indicates that it can provide sufficient Shrub function for cell survival and normal proliferation. In addition, the endosomal distribution of Notch is similar to that in the surrounding wild-type cells, indicating that the endosomal ESCRT function is normal. (B) Co-immunoprecipitation of LGD1 with CHMP4B^redox^. LGD1 co-immunoprecipitates with CHMP4B, indicating that the conformation of refolded CHMP4B enables the interaction with its partner LGD1. The still existing, but significantly reduced amount of precipitated LGD1 in the control sample, after the incubation with denatured CHMP4B, is most likely the result of the spontaneous refolding of CHMP4B under native CoIP conditions. Scale bars: (A) 50 μm; (A’, A”) 10 μm. (A-A”) At least ten wing imaginal discs were analysed for each genotype.

**Additional file 8: Figure S8.** LGD1 binding does not affect the conformation of CHMP4B in solution. (A) Schematic representation of cysteine-based crosslinking assay of CHMP4B. (B) Representative result of the assay performed three times independently. The percentages are the average of all three experiments. Coomassie-Blue staining of non-reducing SDS-PAGE gel of CHMP4B^redox^. In solution, the majority of CHMP4B exists in the faster migrating closed conformation (73,9%). Nevertheless, a significant fraction (26,1%) exists in the slower migrating open conformation (lane 1). As expected, the addition of the reducing DTT (10 mM) abolishes the fraction of CHMP4B^redox^ existing in the closed form (lane 2). The fraction that exists in the closed form can be fixed by addition of Cu^2+^Phenanthroline, which forms intramolecular disulfide bonds between the two existing cysteines in the N- and C-terminus (lane 3). In Lane 4, CHMP4B^redox^ was first incubated with DTT for 20 min. at 37 °C to remove the fraction existing in the closed form. Then, the sample was incubated at room temperature for 30 min. before Cu^2+^Phenanthroline was added to catch the re-emerging fraction of closed form. 29,9% of CHMP4B has again adopted the closed conformation, indicating that CHMP4B is in a dynamic equilibrium between the open and closed conformation. Lanes 5–7: The addition of LGD1 does not affect the conformation of CHMP4B, indicated by the fact that the distribution of closed and open CHMP4B molecules is comparable to corresponding samples without LGD1 (lanes 1, 3, and 4).

## Data Availability

All data generated or analysed during this study are included in this published article and its supplementary information files.
