# Supplementary figures and images for: Lethal (2) giant discs (Lgd)/CC2D1 is required for the full activity of the ESCRT machinery
Source: BMC Biol. 2020 Dec 22;18:200. doi: 10.1186/s12915-020-00933-x (PMC7754597; doi:10.1186/s12915-020-00933-x)

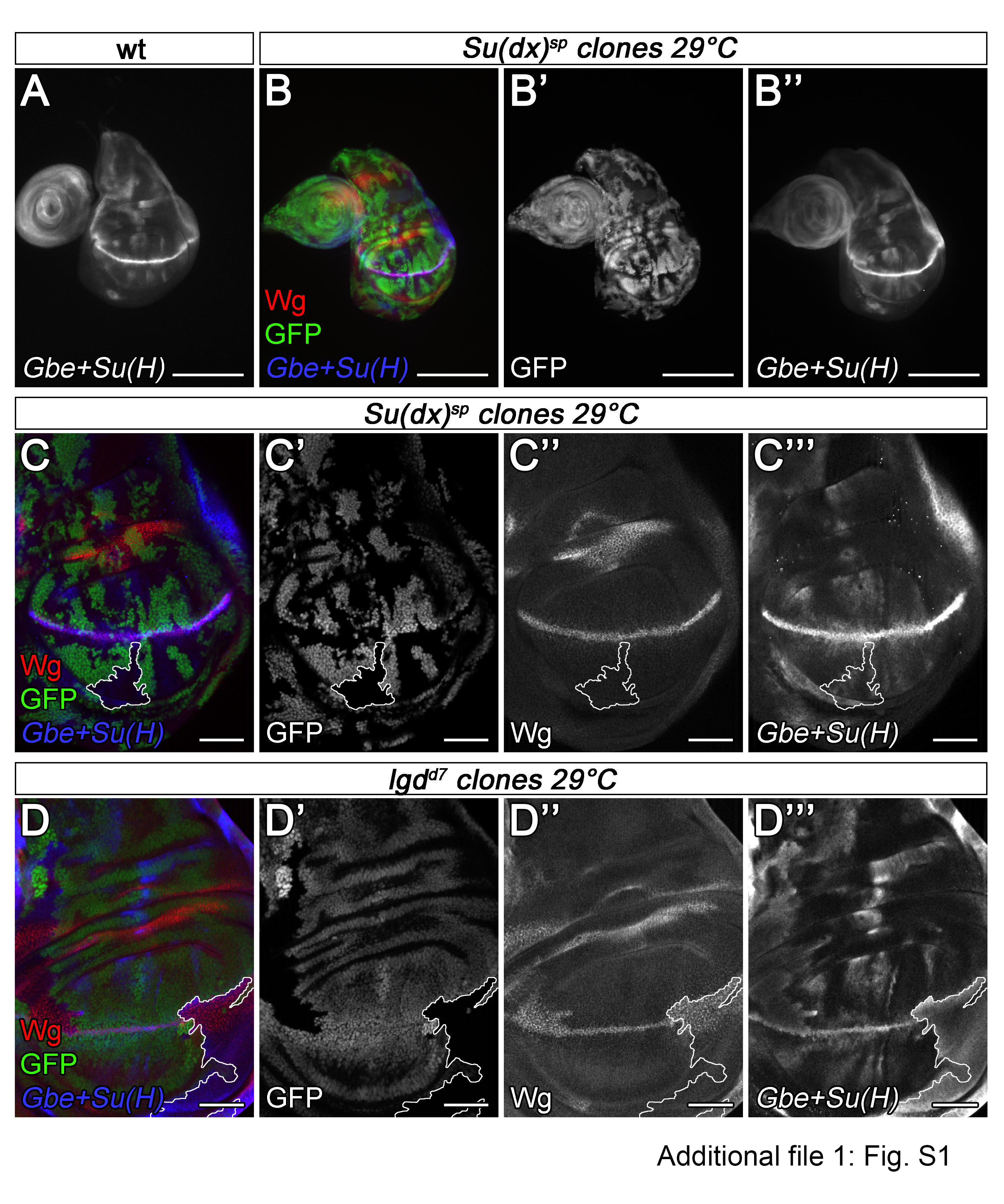

Supplement: Supplementary file 1 — Additional file 1: Figure S1. Clonal analysis of Su(dx). Clones are labelled by the absence of GFP. (A) Expression of the Notch activity reporter Gbe+Su(H) in a control wild-type disc. (B-C”’) A disc bearing Su(dx)sp clones, which is kept on 29 °C to maximise the mutant phenotype. Notch activity is revealed by the expression of Gbe+Su(H). (B-B”) Overview of the disc. (C-C”’) Magnification of the wing area. A large Su(dx)sp mutant clone is outlined in white. No ectopic expression of Gbe+Su(H) is observed. (D-D”’) The wing area of a disc bearing lgd mutant clones, labelled by the absence of GFP. One large lgd mutant clone is outlined in white. The expression of Wg and Gbe+Su(H) is clearly activated ectopically (in all clones). Scale bars: (A-B”) 200 μm; (C-D”’) 50 μm. At least ten wing imaginal discs were analysed for each genotype. [file 12915_2020_933_MOESM1_ESM.tif]

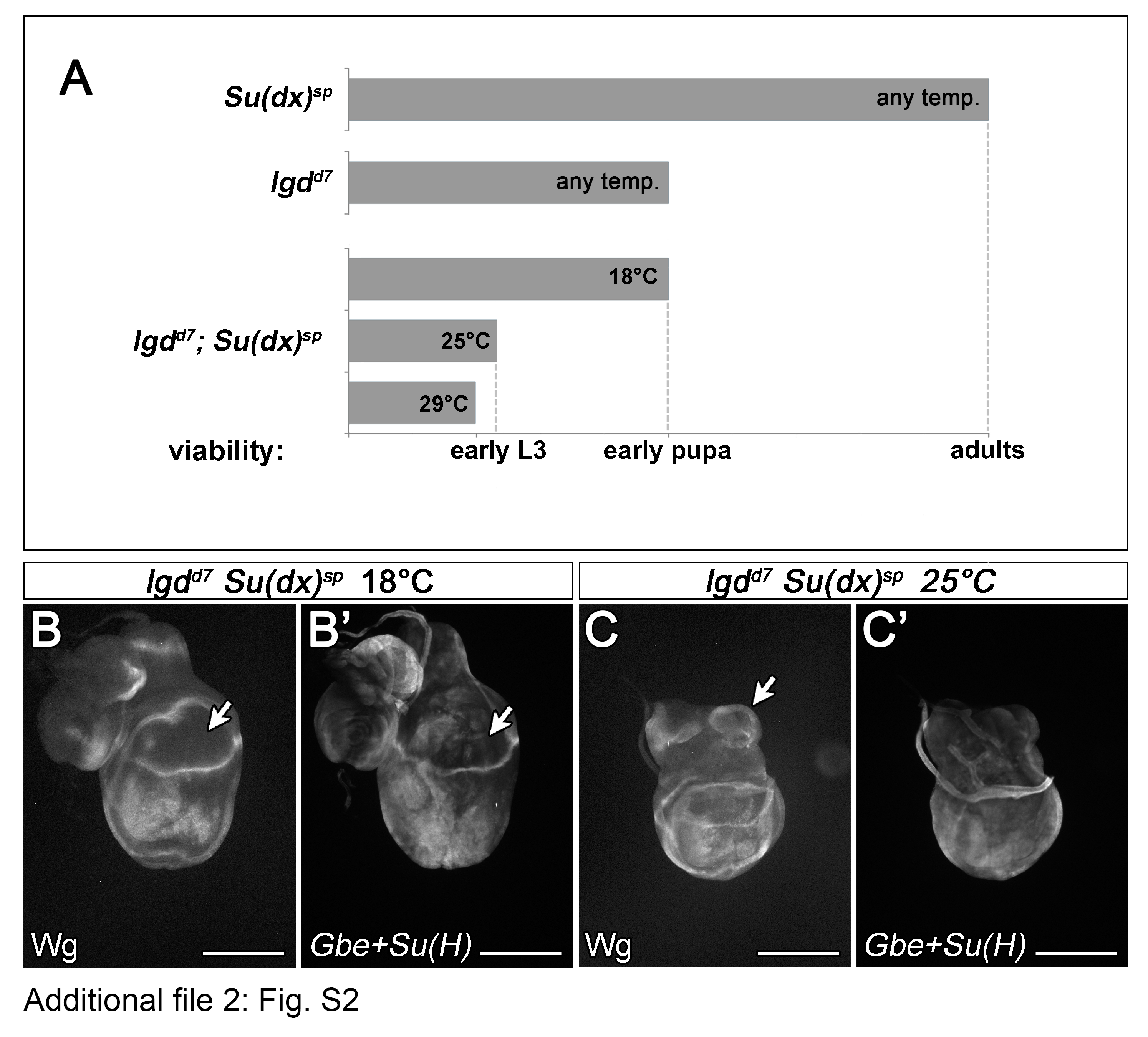

Supplement: Supplementary file 2 — Additional file 2: Figure S2. Genetic interactions between lgd and Su(dx). (A) Time of death of the single and double mutants during development. (B-C’) The phenotype of wing discs of lgd Su(dx) double mutants at 18 and 25 °C, respectively. The arrow in (B, B’) points to the suppression of Notch activation in the dorsal compartment of the wing anlage. The arrow in (C) highlights the formation of an additional winglet often seen upon strong Notch activation. For further information see text. Scale bars: (B-C’) 200 μm. At least ten wing imaginal discs were analysed for each genotype. [file 12915_2020_933_MOESM2_ESM.tif]

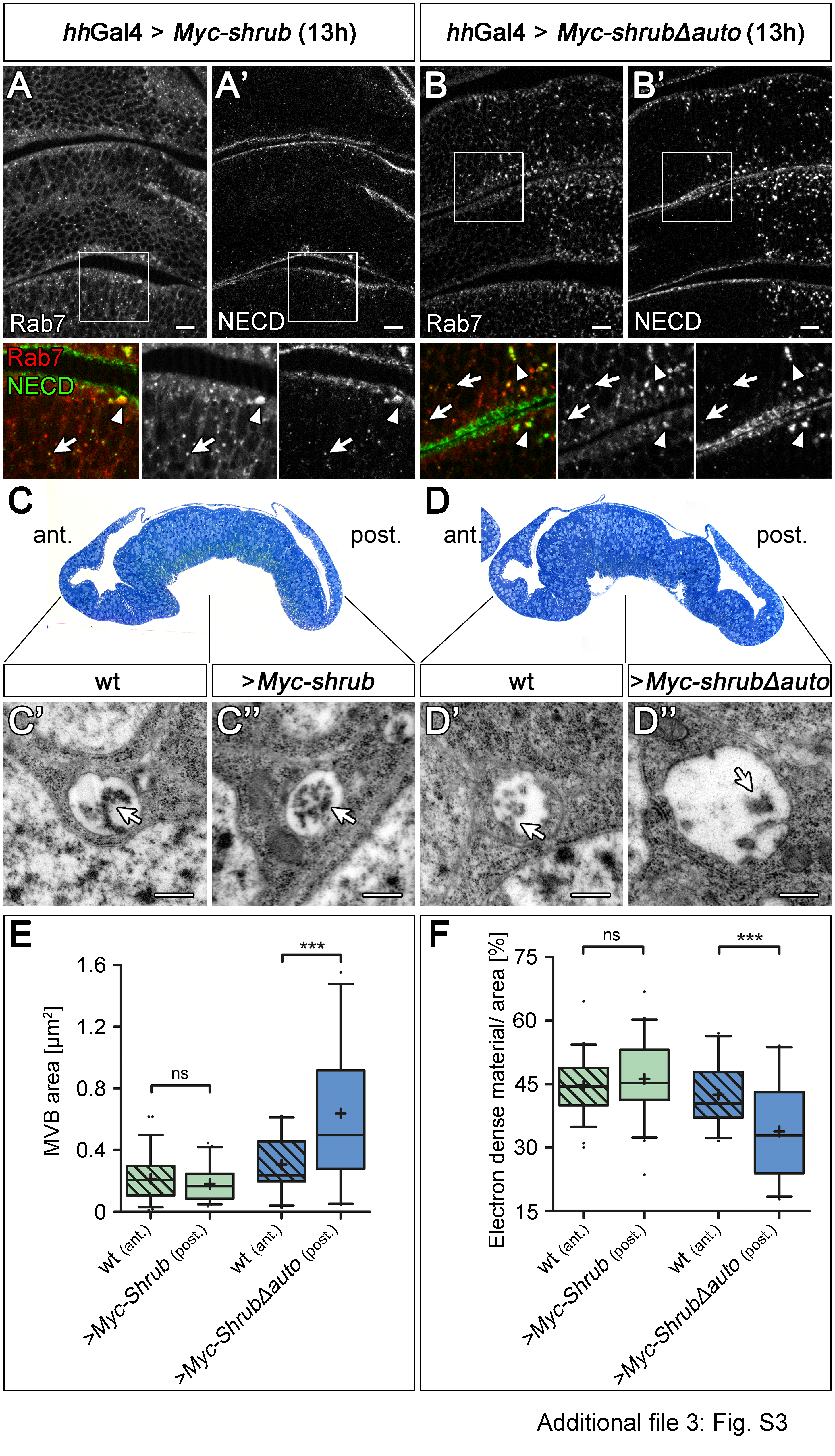

Supplement: Supplementary file 3 — Additional file 3: Figure S3. Analysis of MEs/MVBs of cells expressing Shrub or ShrubΔauto. Comparative analysis of the consequences of expression of Myc-Shrub and Myc- ShrubΔauto. Restricted expression of these variants in the posterior (post.) compartment for 13 h is achieved by using hhGal4 combined with tubGal80ts. (A-B’) In contrast to expression of Myc-Shrub (A-A’), the expression of Myc-ShrubΔauto (B-B’) causes the formation of enlarged Rab7-YFP (tub-Rab7-YFP) and Notch positive MEs. Magnification of the boxed area in (A-B’) explicitly shows that Notch positive structures are MEs (ant. arrows and post. arrowheads). (C-F) TEM analysis of the discs shown in (A, B). (C, D) Transverse semi-sections of analysed wing imaginal discs. (C’-D”) Representative TEM images of the MEs/MVBs of the wild-type anterior (ant.) control cells and posterior cells expressing the Shrub variants. (E) Measurement of the area of the MEs/MVBs. The average area of MEs of Myc-ShrubΔauto expressing cells is significantly increased in comparison to the anterior control cells (blue). However, there is no significant change in the MVB area upon over-expression of full-length Shrub for 13 h (light-green). (F) Pixelwise Quantification of the electron dense material of the MEs/MVBs. Quotient of ILV/ electron dense material per MVB area [%] are collected and plotted in a box plot. The ILV content within MVBs of cells expressing Myc -Shrub is similar to wild-type, whereas it is significantly reduced in MVBs of cells that express Myc-ShrubΔauto. [(E, F) Over-expression of Myc-Shrub (highlighted in light-green): wt control (ant.): n = 52 MVBs, Myc-Shrub (post.): n = 46 MVBs; Over-expression of Myc-ShrubΔauto (highlighted in blue): wt control (ant.): n = 25 MVBs, Myc-ShrubΔauto (post.) n = 33 MVBs; (E) Over-expression of Myc-Shrub (light-green): Mann Whitney test, Two-tailed; Over-expression of Myc-ShrubΔauto (blue): unpaired t-test; (F) Over-expression of Myc-Shrub and of Myc-ShrubΔauto: both unpair [file 12915_2020_933_MOESM3_ESM.tif]

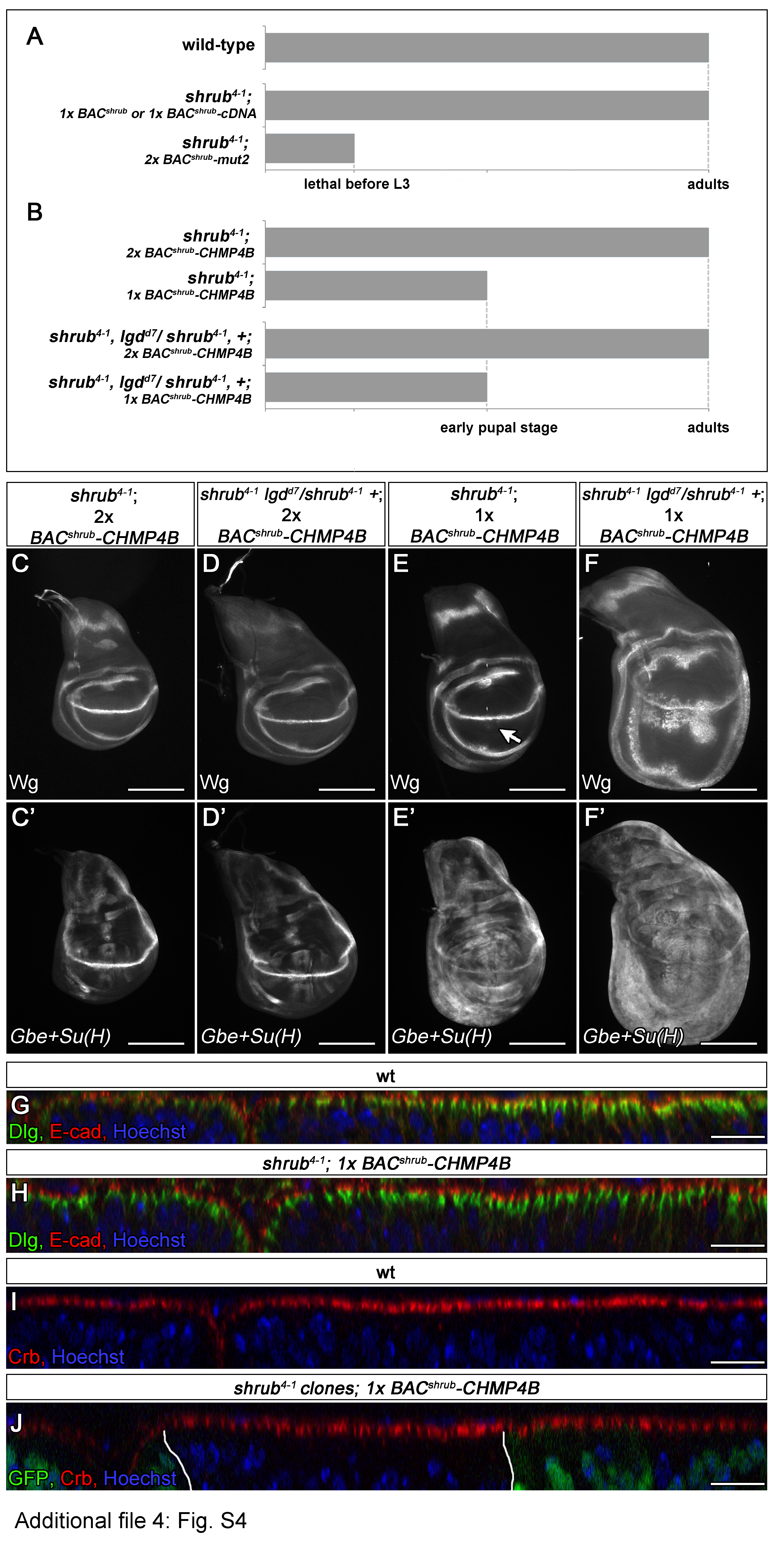

Supplement: Supplementary file 4 — Additional file 4: Figure S4. Rescue of shrub null mutants with BACshrub-CHMP4B. (A, B) Time of death during development of various genotypes rescued by various copies of BACshrub, BACshrub-cDNA or BACshrub-mut2 (A), or BACshrub-CHMP4B (B). (C-F’) Wing imaginal discs of flies rescued with various copies of BACshrub-CHMP4B. (C, C’) The Notch activity detected by Wg or Gbe+Su(H) in presence of two copies BACshrub-CHMP4B is comparable to wild-type, even in lgd heterozygosity (D, D’). (E, E’) A partial rescue is observed if only one copy of BACshrub-CHMP4B is present. A few cells near the D/V-boundary show an ectopic activation of Wg (E, arrow). (E’) Gbe+Su(H) is slightly ectopically expressed. (F, F`) This ectopic activation is enhanced, if one copy of lgd is removed in the partial rescued discs. The observed phenotype resembles that of strong lgd mutants (compare with Fig. 1C, C’). (G-J) Z-stack through the wing pouch of wing imaginal discs: Comparative Analysis of the apicobasal polarity of wild-type wings discs and shrub mutant discs partially rescued with one copy of BACshrub-CHMP4B. (J) Induction of shrub mutants clones in presence of one copy of BACshrub-CHMP4B. Mutants clones are labelled by the absence of GFP. (G-J) The apicobasal polarity is unaffected in comparison to the wild-type, indicated by the normal localisation of E-cad, Dlg and Crb. Scale bars: (C-F’) 200 μm; (G, H) 50 μm; (G-J) 10 μm. (C-J) At least ten wing imaginal discs were analysed for each genotype. [file 12915_2020_933_MOESM4_ESM.tif]

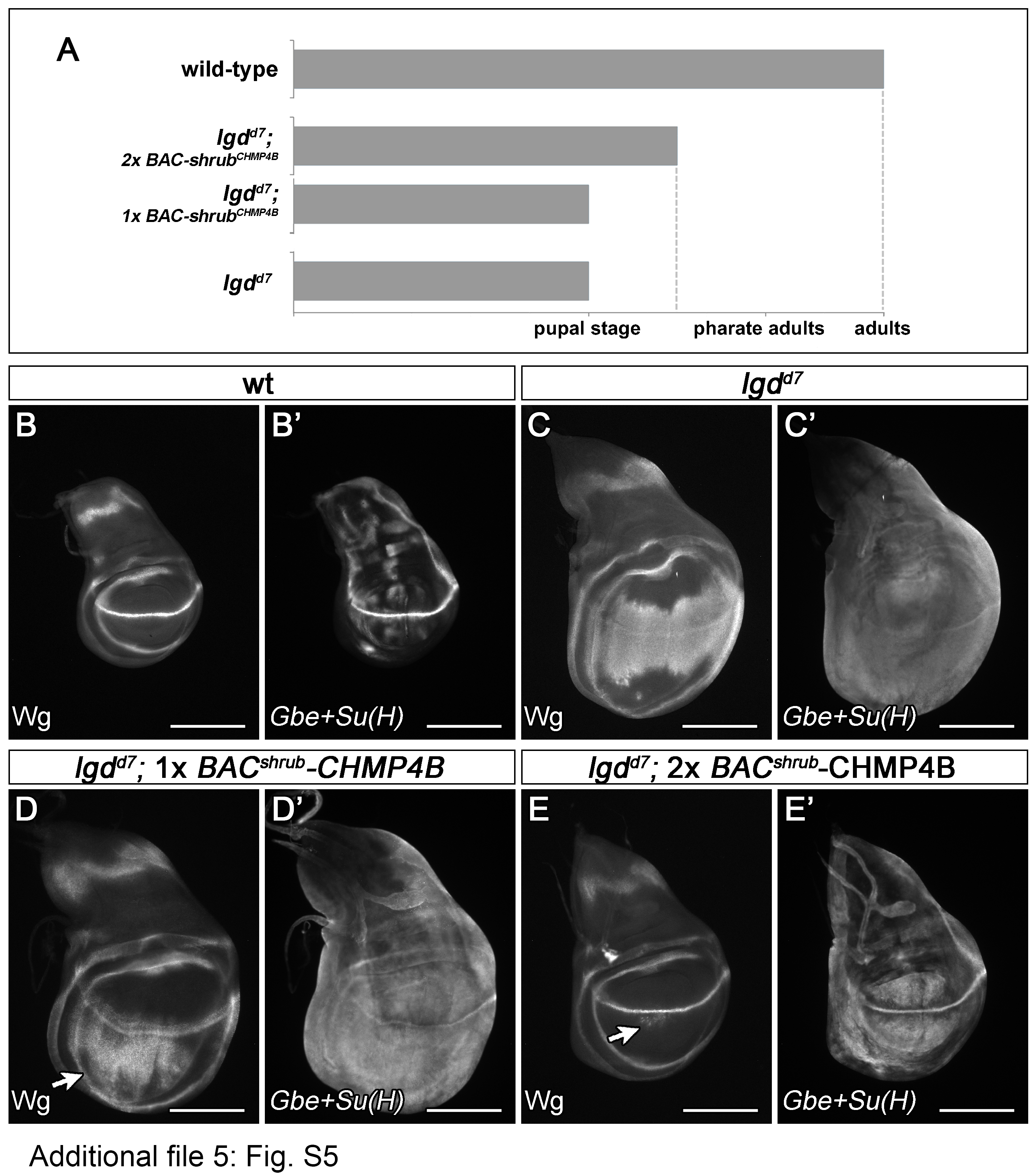

Supplement: Supplementary file 5 — Additional file 5: Figure S5. Addition of copies BAC-shrubCHMP4B partially rescues lgd mutant flies. (A) Summary of the time of death of lgd mutant flies rescued with one or two copies of BACshrub-CHMP4B. (B-E`) The expression of Notch targets in the corresponding wing imaginal discs reveals that the degree of rescue of lgd mutants is proportional to the number of copies of BACshrub-CHMP4B present in the genome. Scale bars: (B-E’) 200 μm. At least ten wing imaginal discs were analysed for each genotype. [file 12915_2020_933_MOESM5_ESM.tif]

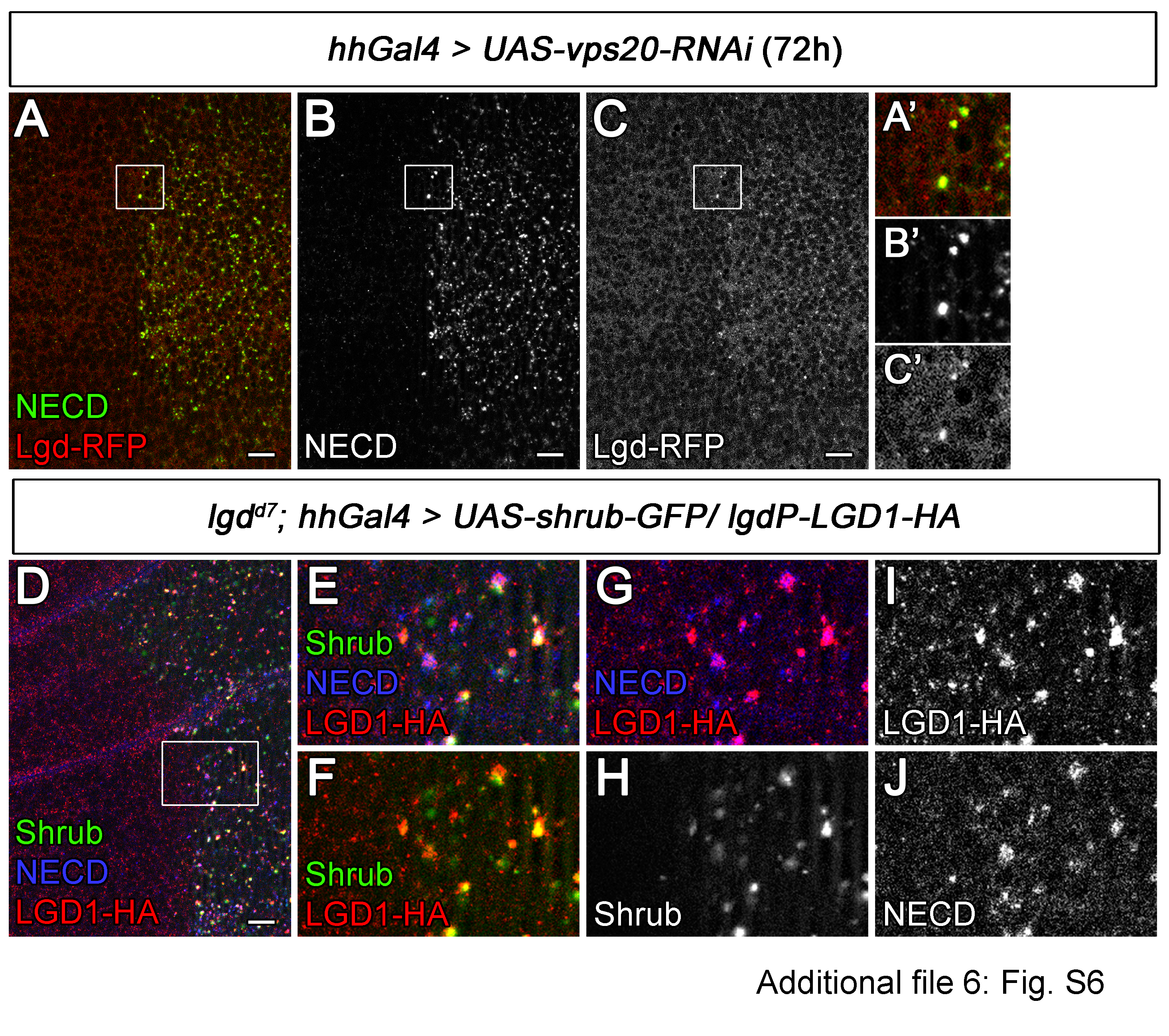

Supplement: Supplementary file 6 — Additional file 6: Figure S6. Lgd cycles between the cytosol and the LM of the ME. (A-C’) Depletion of the function of vps20 in the posterior compartment of the wing discs results in the formation of strongly enlarged Notch positive MEs, but in only weak accumulation of Lgd-RFP on the LM. Magnification of the area boxed in (A-C) is shown in (A’-C’). (D-J) Expression of Shrub-GFP in the posterior compartment of a lgd mutant wing disc rescued with LGD1 expressed under control of the lgd promoter results in the accumulation of LGD1-HA on enlarged Notch positive MEs. This shows that also LGD1 can be recruited to the ME by Shrub. (E-J) Magnification of the region boxed in (D) is shown in (E-J). Scale bars: (A-D) 10 μm. At least ten wing imaginal discs were analysed for each genotype. [file 12915_2020_933_MOESM6_ESM.tif]

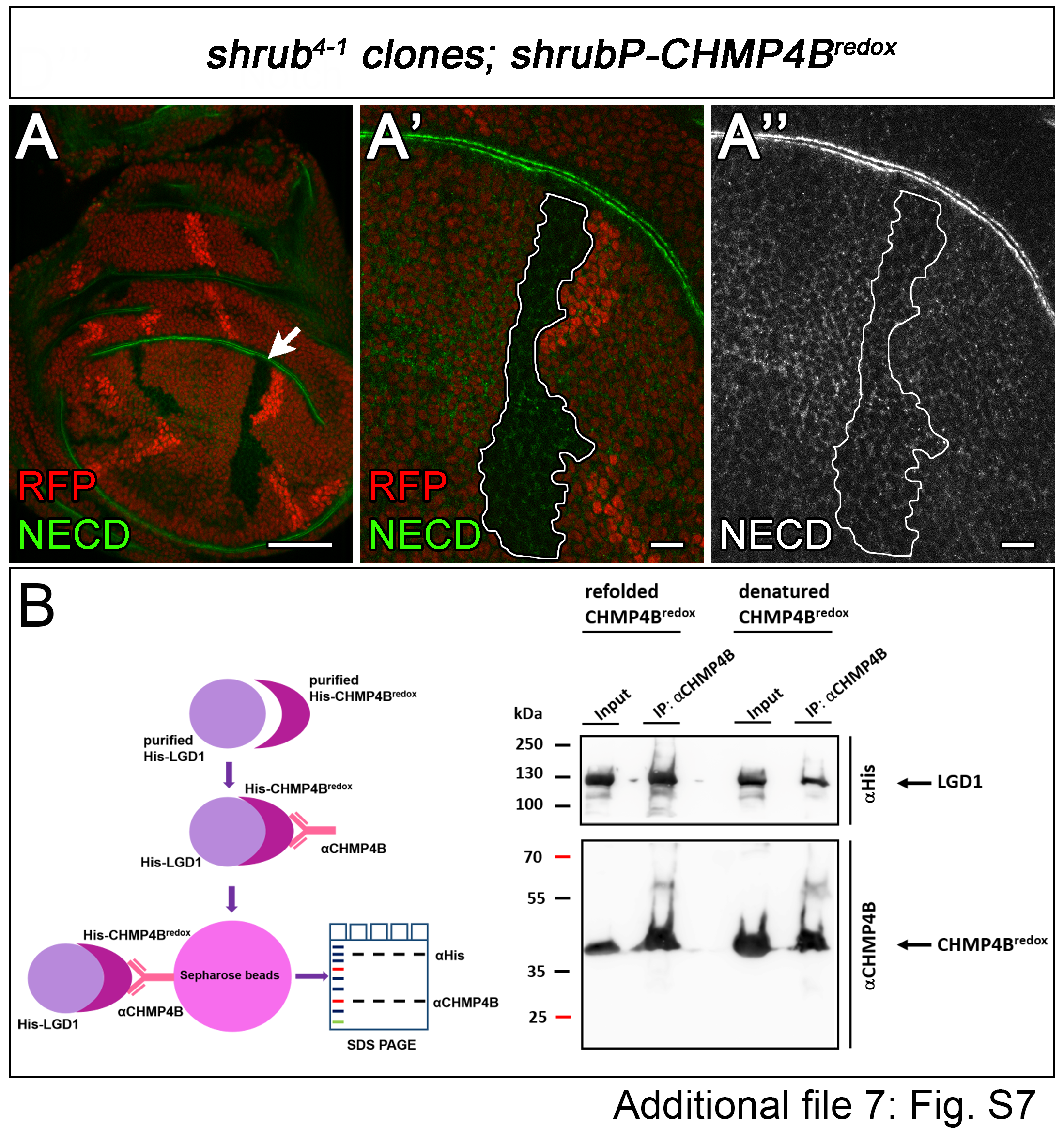

Supplement: Supplementary file 7 — Additional file 7: Figure S7. Testing the functionality of shrubP-CHMP4Bredox. (A-A”) Induction of shrub mutant clones in the presence of one copy of shrubP-CHMP4Bredox in the genome. Clones are labelled by the absence of RFP and outlined with the white line in (A’, A”). The arrow in (A) points to a large clone shown at higher magnification in (A’, A”). Loss of shrub function in wing disc cells is normally cell lethal (see Fig. 5A–E). The presence of CHMP4Bredox prevents death of the shrub mutant cells and leads to a normal appearance of the MEs in the mutant cells. This indicates that it can provide sufficient Shrub function for cell survival and normal proliferation. In addition, the endosomal distribution of Notch is similar to that in the surrounding wild-type cells, indicating that the endosomal ESCRT function is normal. (B) Co-immunoprecipitation of LGD1 with CHMP4Bredox. LGD1 co-immunoprecipitates with CHMP4B, indicating that the conformation of refolded CHMP4B enables the interaction with its partner LGD1. The still existing, but significantly reduced amount of precipitated LGD1 in the control sample, after the incubation with denatured CHMP4B, is most likely the result of the spontaneous refolding of CHMP4B under native CoIP conditions. Scale bars: (A) 50 μm; (A’, A”) 10 μm. (A-A”) At least ten wing imaginal discs were analysed for each genotype. [file 12915_2020_933_MOESM7_ESM.tif]

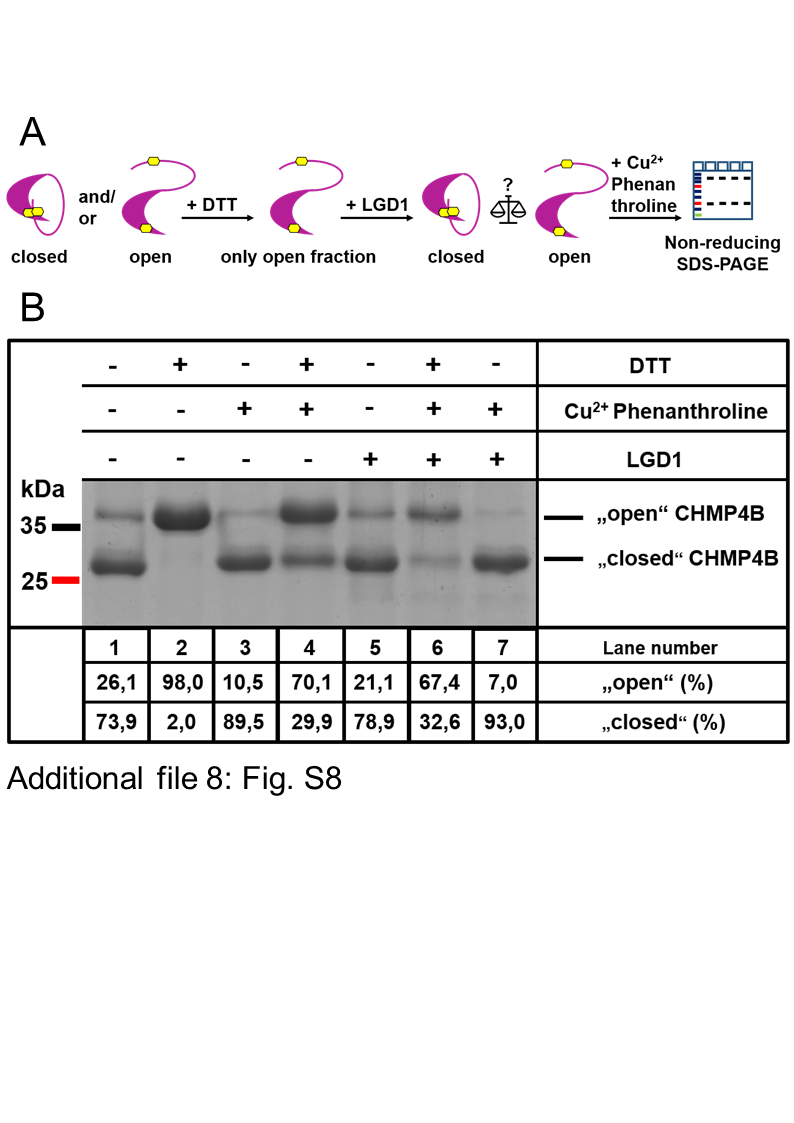

Supplement: Supplementary file 8 — Additional file 8: Figure S8. LGD1 binding does not affect the conformation of CHMP4B in solution. (A) Schematic representation of cysteine-based crosslinking assay of CHMP4B. (B) Representative result of the assay performed three times independently. The percentages are the average of all three experiments. Coomassie-Blue staining of non-reducing SDS-PAGE gel of CHMP4Bredox. In solution, the majority of CHMP4B exists in the faster migrating closed conformation (73,9%). Nevertheless, a significant fraction (26,1%) exists in the slower migrating open conformation (lane 1). As expected, the addition of the reducing DTT (10 mM) abolishes the fraction of CHMP4Bredox existing in the closed form (lane 2). The fraction that exists in the closed form can be fixed by addition of Cu2+Phenanthroline, which forms intramolecular disulfide bonds between the two existing cysteines in the N- and C-terminus (lane 3). In Lane 4, CHMP4Bredox was first incubated with DTT for 20 min. at 37 °C to remove the fraction existing in the closed form. Then, the sample was incubated at room temperature for 30 min. before Cu2+Phenanthroline was added to catch the re-emerging fraction of closed form. 29,9% of CHMP4B has again adopted the closed conformation, indicating that CHMP4B is in a dynamic equilibrium between the open and closed conformation. Lanes 5–7: The addition of LGD1 does not affect the conformation of CHMP4B, indicated by the fact that the distribution of closed and open CHMP4B molecules is comparable to corresponding samples without LGD1 (lanes 1, 3, and 4). [file 12915_2020_933_MOESM8_ESM.tif]
